# Ten Steps to Organize a Virtual Scientific Symposium and Engage Your Global Audience

**DOI:** 10.1002/gch2.202200005

**Published:** 2022-06-26

**Authors:** Jiye Son, Jasmine Sabio, Ankit Jain, Rein V. Ulijn

**Affiliations:** ^1^ Nanoscience Initiative Advanced Science Research Center at The Graduate Center City University of New York 85 St. Nicholas Terrace, New York NY 10031 USA; ^2^ Department of Chemistry Hunter College City University of New York 695 Park Avenue, New York NY 10065 USA; ^3^ Ph.D. Programs in Chemistry and Biochemistry The Graduate Center of the City University of New York 365 Fifth Avenue, New York NY 10016 USA

**Keywords:** online scientific symposia, planning guide, Twitter poster presentations, virtual conferences, Zoom webinar

## Abstract

The paper describes guidelines for the planning, organization, and successful execution of virtual, global scientific conferences for global audiences. The guidelines are based on experience and lessons learned during the organization of the 3‐day 2020 Virtual Systems Chemistry Symposium hosted on Zoom webinar and Twitter, held on May 2020 with over 1000 registered participants from 46 different countries.

## Graphical abstract

This planning guide outlines ten steps that academic organizers can follow to successfully organize and execute virtual, global scientific symposia for global audiences. The authors have also included instructional guides, videos, and PowerPoint slides that can be used as templates or inspiration for hosting virtual symposia with oral presentations on Zoom webinar and poster presentations on Twitter.

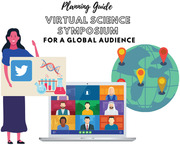

## 1 Introduction

Planning any scientific symposium, whether in‐person or virtual, takes tremendous teamwork and meticulous planning from the organizers to ensure a successful event. In early 2020 when all in‐person events were cancelled due to the COVID‐19 pandemic, many organizers, including our team, had to quickly learn how to transition the in‐person events into the virtual platform. We had to navigate a number of learning curves with few points of reference or guidance available. Considerations went much beyond selection of speakers, agreeing daily duration and time zone to enable global participation, agreeing on duration of talks and discussion sessions, which platform to host the speaker sessions, the poster presentations, the networking discussions, social aspects, and the list goes on—and all of this at a time where none of the organizers nor the participants were very familiar with the virtual platform! After 18 months of near absence of in‐person events, many lessons have been learned and the competitive advantages of hosting virtual conferences have been recognized.^[^ ^1^ ^]^ These include low barriers to attendance (travel, finance), easy access, flexibility, the ability to host meetings across many time zones, which have enormously increased the number and diversity of conference attendees including students and postdocs by 344%, female participation by 253% and 700% increase in participation for gender queer scientists.^[^ ^2^ ^]^ Another notable advantage is the vastly reduced environmental impact, which can reduce the carbon footprint by 94% and energy use by 90% in comparison to in‐person conferences.^[^ ^3^ ^]^

Another unexpected advantage of the virtual experience was a sense of closeness with speakers who would otherwise be presenting from a distant lectern in front of a large audience in a traditional in‐person meeting. By viewing the speakers present in real time (as opposed to watching a recorded presentations) on screen, many attendees reported a sense of intimacy even with highly esteemed professors who are typically less accessible. The speakers often adjusted their style as if they were speaking directly to an individual person. Therefore, while there is no substitute for in‐person interactions in groups, virtual conferences are here to stay and will continue to enrich the conference scene alongside more traditional in‐person conferences.

The permanence and the demand for virtual conferences are backed by profitable businesses who organize virtual events, often at substantial cost. However, many academic institutions will still prefer to organize these events themselves, and we have experienced that with the right, committed team and some careful planning, a successful global event can be organized with relative ease. Here, we present a detailed “how‐to” process in ten steps for planning and executing a live, large, global, virtual scientific symposium. Each of the steps outlines our process, the resources and template guides that we have prepared along the way, and our recommendations for future planning based on the turnout, feedback, and lessons learned.

This report is mainly based on our experience as lead organizers of the 2020 Virtual Systems Chemistry Symposium,^[^ ^4^ ^]^ held on May 2020 with over 1000 registered participants from 46 different countries and across 14 time zones. The symposium was co‐chaired by researchers from six academic institutions across the US, Europe, and the Middle East: Rebecca Schulman, Johns Hopkins University, USA; David Lynn, Emory University, USA; Gonen Ashkenasy, Ben‐Gurion University of the Negev, Israel; Rafal Klajn, Weizmann Institute of Science, Israel; Sijbren Otto, University of Groningen, The Netherlands; and led by a team of staff, students, and faculty at the CUNY Advanced Science Research Center (CUNY ASRC), USA. This 3‐day virtual symposium included three keynote speakers and 12 invited speakers throughout the six interactive talk sessions, a Twitter poster session with 115 posters across five sub‐sections and eight poster winners who were selected to give short talks on the last day, and social discussion chat rooms. After the conference, the scientific and intellectual content of the symposium was published in an article which was co‐written by four early career session speakers in an article titled “Complexity Emerges From Chemistry” and published in Nature Chemistry.^[^ ^5^ ^]^

After our event, we were approached on several occasions for advice and guidance where we were glad to share our resources. This triggered us to seek publication of our materials, so that these can be widely shared and used by other virtual conference organizers. By sharing our “know‐hows” and learnings from the 2020 Virtual Systems Chemistry Symposium and other virtual events since then, we hope to lower the energy barrier for organizers who plan virtual events and improve the overall experience for all the participants. We hope that the guides and resources that we share here will be leveraged as templates or inspiration and help organizers plan and execute a successful virtual event.

## 2 Step 1: Establish a Timeline

**Figure 1:**
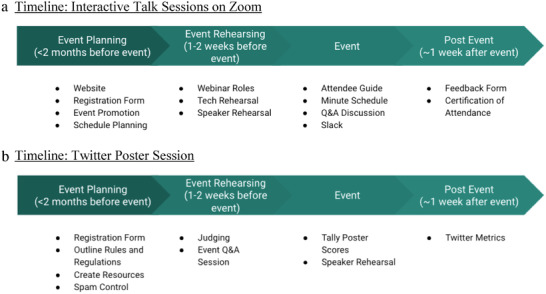
Schematic of a timeline for planning and executing a virtual event that include a) interactive talk sessions on Zoom and b) Twitter poster session.

When an event is planned at a short notice, considering how much needs to be done can be overwhelming. Conversely, when an event is planned too far in advance, it is easy to lose focus and momentum and not progress until there is only a short amount of time left. A timeline can help the organizers stay on track and accomplish the tasks at the opportune timing. Therefore, our first recommendation is to establish a timeline that makes sense for the size and scale of your virtual event. Below is an example of a general timeline we used to plan for the 2020 Virtual Systems Chemistry Symposium, which began 2 months prior to the event (**Figure** **1**). If possible, we recommend sending out requests to invited speakers 6–12 months in advance, followed by creation if a Twitter handle and promoting through a “save the date” or first announcement once these speakers have committed. Detailed planning can then start 2 months prior to the event.

## 3 Step 2: Recruit the Organizing Team

**Figure 2:**
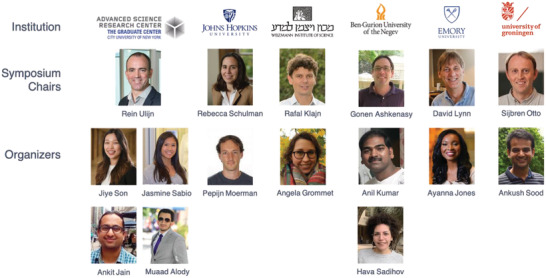
Main organizers from six international institutions. Photos of organizers presented with their permission, and logos of their institutes reproduced with those institutes’ permission.

Our symposium was co‐chaired by research leaders from 6 academic institutions from different time zones and countries with a shared interest in systems chemistry. As the leading organizing institution, the CUNY ASRC led with four organizers (three staff members and one postdoc). There were additional six Ph.D. students and postdocs from the five co‐organizing institutions (**Figure** **2**).

To organize an inclusive event that catered to the global systems chemistry audience, it was important to have representation and significant contributions and from each of the co‐chairs’ institutions located in different countries and time zones. Our multi‐institutional, co‐organization approach ensured event promotion through our collective networks. In addition, we were better placed to consider time zones and local preferences of the global audience when selecting the appropriate times and days for the conference. We were able to quickly solve unexpected technical issues. For example, we faced problems in delivery of email blasts from our US public university accounts to international university email domains in some instances, this was easily rectified by sending out from a different email address. By working closely with co‐organizers from the different institutions, we were able to reach a broader audience through amplified use of social media for advertising and receive real‐time feedback on the effectiveness or issues with the event promotion at a local level.

## 4 Step 3: Choose the Platform

There is now a plethora of software applications to host video conferences (Zoom Meeting and Webinar, Cisco Webex Meeting, Google Meet, Adobe Connect, Microsoft Teams) with applications to assist in virtual networking (Slack, Gather.town, Discord, Twitter) and launch live‐time data collection (Zoom polls, Mentimeter). Choose the platform that is appropriate for your event.

Our interactive talk sessions were hosted using Zoom webinar, poster sessions were hosted on Twitter, and the social chat rooms were created on Slack Channels. Email blasts were sent out using Pardot by Salesforce, and registration and feedback surveys were created on Formstack. Event webpage was created on CUNY ASRC's website domain on WordPress. Marketing materials were created on Spark Adobe, Canva, and PowerPoint. During the live event, the organizers used WhatsApp to communicate and Google Docs to record information live‐time (number of participants, Zoom poll results, and unanswered questions).

## 5 Step 4: Develop an Engaging Event Program; Interactive Talk and Twitter Poster Sessions

### 5.1 Interactive Talk Sessions

Over the 3 days, we scheduled one keynote speaker to start each day of the event (total of three keynote speakers). There were 12 speakers that we grouped into six sessions over the 3 days (two speakers per session). Generally, we found that the audience's attention spans are shorter in virtual meetings,^[^ ^6^ ^]^ so we chose to have short speaker slots (15 min) and longer discussions (10 min). Instead of giving an overview of their work, we asked speakers to focus on one or two recent discoveries. This approach provided an insight into the intricate details of a recently published, or to be published work and was well‐received by the audience who appreciated a fresh perspective, especially from the prominent keynote speakers. The session chairs were directed to introduce the session topic and to connect the talks by asking questions that could be answered by multiple speakers. This strategy provided additional context for each session and tied into the overall theme of the symposium. We also decided to include a current, topical session on “Systems Chemistry and the Coronavirus Crisis” to highlight the immediate significance and application of the research field. Our finalized program can be found on our website with hyperlinks to the talk abstracts.^[^ ^7^ ^]^ A sample of Day 1's program is also available in Form S11, Supporting Information.

### 5.2 Twitter Poster Sessions

Poster session topics corresponded with the interactive oral presentation sessions. @SysChem20 handle was used, which quickly gained as many as 955 followers (as of October 2021) and hosted a total of 115 Twitter posters. The poster sessions started 24 h before the first day (Day 1) of the oral presentation sessions and remained open for 48 h (Days 1–2), then ended 24 h before the last day (Day 3). Taking into consideration the different time zones, we scheduled the poster sessions to start a full 24 h in advance of the symposium to allow participants plenty of time to browse and interact with the presenters through the reply, retweet, and like functions on Twitter. Participants were also given the option to continue lengthier discussions on Slack in the corresponding session topic channels. The poster session ended a day prior to the end of the symposium for the organizers to tally up poster scores (see Section 9), select winners, and prepare poster winners for their talks on Day 3. Winners from the poster sessions were selected to present on Day 3 of the symposium which was student led with the post‐docs from the organizer committee moderating the talks. Two winners from those who presented talks on Day 3 of the symposium were awarded full registration support and the opportunity to present a short talk for Systems Chemistry Gordon Research Conference in 2022. A full list of the Twitter Presentations can be found on our website with hyperlinks to the posters on Twitter.^[^ ^8^ ^]^ A sample of session “Dynamic Information of Molecular Assemblies” is also available in Form S12, Supporting Information.

## 6 Step 5: Event Promotion

Before promoting the event, a branding style and graphic were chosen which were then used to create a website, Twitter account, and registration page with recurring brand aesthetics. To promote our event, we used Twitter and email blasts to advertise to our co‐chairs and organizer's network of colleagues and reached out to editors of scientific journals and funding body program managers in the field. Some of the journal editors were also asked to act as poster judges and increased our visibility by retweeting our promotion tweets (see Section 9.5). Updates on event details and confirmed speakers were frequently tweeted and posted on the website in real‐time.

### 6.1 Branding

**Figure 3:**
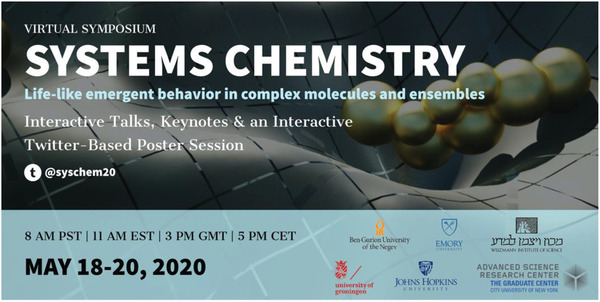
Main graphic for event promotion. Variations of this graphic were used to fit different media format.

We created an aesthetic graphic that can be used consistently throughout the event promotion to create a coherent brand image for the event. The main graphic featured the title and brief description of the event, dates, times, logos of the organizing institutions, and a Twitter handle where more information can be found (**Figure** **3**). Variations of the main graphic were used to fit different media (email blasts, Twitter, web page) by adjusting the size and simplifying the content to fit banners, flyers, etc. while consistently delivering the same brand imaging.

### 6.2 Event Webpage

Our event webpage served as a central resource for program, registration, and news items related to the event. During the early planning stage, the webpage can be a simple landing page that is at least appropriately branded, and display the title, date, and brief overview of the event. Even with minimal information, it is important to have a finished landing page with aesthetic branding to promote the event on social media, email blasts, and to reliably direct potential participants and attendees to the webpage more information. We updated the webpage frequently as the planning stage developed and populated the page with registration link, confirmed speakers, agenda, guidelines, etc. The event webpage was created on CUNY ASRC's website domain on WordPress.^[^ ^4^ ^]^

### 6.3 Twitter

In the early planning stage, the Twitter page and bio displayed the event graphic, event webpage link, and date of the event. As the event planning progressed, the event registration link was added to the bio and frequent tweets were posted to announce new confirmed speakers. We collaborated with Nature Nanotechnology's senior editor, Dr. Alberto Moscatelli, who created “3‐minute Science” videos to introduce some of our speakers including Dr. Schwille^[^ ^9^ ^]^ and Dr. Thordarson^[^ ^10^ ^]^ on Twitter. When possible, tweets should always be posted with an accompanying image to increase average number of clicks and likes and increase the chance of retweets by 150%.^[^ ^11^ ^]^ Changing the Twitter handle to include the specific year of the symposium is a good way to organize poster presentations which can be easily found and sorted from the past symposium's presentations. This strategy is also beneficial when follow up events are organized. The twitter handle was changed to @SysChem21 for the 2021 Virtual Systems Chemistry Symposium led by Thomas Hermans (University of Strasbourg, France) and co‐organized by CUNY ASRC and 4 other institutions.^[^ ^12^ ^]^

### 6.4 Email Campaign

After the webpage, Twitter account, and registration page were set up, our event was promoted through email blasts. Email blasts were reserved for big announcements such as a finalized speaker list, call for poster abstracts, and Zoom webinar access credentials. Frequent announcements, relevant news items and updates were posted on Twitter.

## 7 Step 6: Registration

The event was free to register and only open to registered attendees. Registration was closed approximately 2 weeks before the event because of the big volume of registrants and our limited capacity of 1000 registered attendees that could be supported by our university's Zoom webinar account. Individual email requests for late registration were accepted since we expected actual attendance to be about 50% of those who registered, which turned out to be close to the actual number of attendees for our event (see Section 11.1).

**Figure 4:**
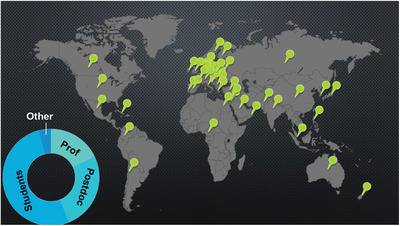
Total registered: 977; Demographic: 58% students, 26% postdoc, 18% professors, 4% other from 48 countries and all continents except Antarctica.

The registration link was created through Formstack. Using Formstack, we could collect more detailed data from the registrants including affiliation, location and title, pre‐assess interest in session topics and poster presentations, and ask to sign‐up for future marketing emails from CUNY ASRC (**Figure** **4**). The collected data gave us an understanding of our audience demographic and increased the number of poster presenters by sending separate reminder emails to registrants who marked their interest in presenting posters. The event registration form can be found in Form S7, Supporting Information.

The poster sessions were open to graduate students, post‐docs, and faculty members who registered for the symposium event. Eligible registrants were asked if they were interested in presenting a poster and asked to rank their preferable session topics of interest. Based on the number of interested presenters, we divided up the presenters as evenly as possible among each session topic. Each topic was assigned a corresponding hashtag (i.e., #name‐of‐session) for their session. Poster session registration form can be found in Form S8, Supporting Information.

## 8 Step 7: Roles and Guides for Interactive Oral Presentation Sessions

The roles for the interactive sessions were defined to fit the capabilities of roles that are offered on Zoom webinar. On Zoom webinar, people can be assigned as host (only one person), co‐host (can be multiple people), panelist, and attendee. See Zoom's info on roles in a webinar.^[^ ^13^ ^]^ Multiple roles were orchestrated together per session. Technical rehearsals were held for session chairs, Q/A and chat moderators, invited speakers, and poster winner speakers to explain their roles and to practice using the Zoom webinar features. Guides were provided for each of the roles to clearly define expectations and code of conduct.

### 8.1 Webinar Host (Zoom Host)

One host is required to open and close the webinar with stable internet connection and assist in configuring webinar settings. Our host was an IT staff member who was not involved in orchestrating the event. Through future events, we found that it is best to have a host who can also carry out the role of a co‐host described below to minimize the number screens displayed during the webinar.

### 8.2 Webinar Co‐Host (Zoom Co‐Hosts)

A minimum of two co‐hosts are required with easy communication to one another outside of Zoom (we used WhatsApp and Google Docs to record event data). Roles for co‐host 1 included screen sharing slides during session/speaker introductions and breaks, announcing housekeeping rules, and recording unanswered questions during discussion. Roles for co‐host 2 included coordinating and moving chairs/speakers/moderators to and from *Zoom participants* to *Zoom panelists*, spotlight speaker, alert speakers of 5 min time‐up, launch Zoom polls, and record number of participants per speaker.

### 8.3 Speakers (Zoom Panelist)

For our event, there were two speakers per session, each with 15 min talk presentations followed by 10 min Q/A and discussions led by session chairs. Rehearsals were set up for each invited speaker individually to discuss event details and practice screen sharing (at the time, most academics were unfamiliar with Zoom). Speaker guide can be found in Form S1, Supporting Information. Rehearsals were also set up for poster winner speakers at the end of Day 2 to practice screen sharing and discuss event details. Same guide was used for all speakers.

### 8.4 Session Chairs (Zoom Panelist)

For each session, there was one co‐chair who presented a brief overview of the session topic, introduced the speakers, and led the Q/A and discussions by reading out loud the posted questions in *Zoom question box* or prompting *attendees* to unmute and ask questions directly. Session chair guide can be found in Form S2, Supporting Information.

### 8.5 Q/A Moderators (Zoom Panelist)

There were two moderators per session who monitored the *Zoom question box* to “clear” questions that have been answered and to “dismiss” duplicate questions. See Zoom's info on using Q/A as the webinar host.^[^ ^14^ ^]^ We outlined the roles of the Q/A and chat moderators and shared a guide with the moderators (student/post‐doc organizers). Since most academics are now familiar and comfortable using the Zoom functions, the Q/A moderator is unnecessary if the session chair can simultaneously monitor the *question box* with ease. Moderator guide can be found in Form S3, Supporting Information.

### 8.6 Chat Moderators (Zoom Attendee)

There were two chat moderators per session who monitored the *Zoom chat box* and assisted participants with technical troubleshooting. At the end of a session, a chat moderator would submit a break into the chat thread and type in “THIS IS THE END OF SESSION 1; ALL FURTHER COMMENTS WILL BE FOR SESSION 2.” We outlined the roles of the Q/A and chat moderators and shared a guide with the moderators (student/post‐doc organizers). We recommend having at least one or more chat moderator depending on the number of expected audience and chat engagement from the audience. Moderator guide can be found in Form S3, Supporting Information.

### 8.7 Live Tweeter (Zoom Attendee)

We assigned one live Tweeter per session to post live on Twitter with speaker start with screenshot images and speaker end posts, and like, retweet, and reply on mentioned tweets.

### 8.8 Participants/Audience (Zoom Attendee)

A participant guide was created to instruct attendees on how to navigate on Zoom webinar, Slack channels and included our Rules of Conduct. Housekeeping rules were repeated at the beginning of each session and reminders to check out Twitter poster presentations or Slack channel were repeated at the end of each session. Participant guide can be found in Form S4, Supporting Information.

## 9 Step 8: Roles and Guides for Twitter Poster Sessions

Roles for the Twitter presentation session were designated across 20 Twitter spam patrol volunteers, 24 poster judges, and six poster winner talk judges.

### 9.1 Rules and Resources for Creating Twitter Account and Posters

Event guidelines, judging criteria, poster guidelines, and rules of conduct were agreed upon among the organizers. Past examples from Royal Society of Chemistry were adapted with permission. Resources were collected and created to ease the onboarding of new Twitter users and virtual poster presenters. Original instructional videos on how to create a Twitter account, how to attend a poster event on Twitter, how to create a GIF, and how to report spam on Twitter were made and posted on YouTube.^[^ ^15^ ^]^ All of the information for Twitter poster presenters can be found on our website with hyperlinks to the videos and external resources.^[^ ^16^ ^]^ A sample of the information page is also available in Form S13, Supporting Information.

### 9.2 Frequently Asked Questions Session

A session was held for poster presenters to ask questions about the poster session on a live Zoom meeting. Questions from the meeting were recorded and posted on our website as frequently asked questions (FAQ) for future reference.^[^ ^16^ ^]^ The FAQ video can be found on YouTube.^[^ ^17^ ^]^

### 9.3 Twitter Spam Patrol Volunteers

20 volunteers located in different time zones followed the @SysChem20 handle and poster hashtags to monitor inappropriate use of the handle or spam bots. Only one suspicious incident was reported where a registered event participant was mistaken for a spam bot due to a handle name that was similar to @SysChem20.

### 9.4 Poster Judging Criteria and Judges

A scoring rubric was created to judge posters with quantifiable scores. One poster winner was selected from each topic category. The judging criteria included 1) research originality, 2) potential impact, 3) comprehensiveness, 4) informative nature, 5) self‐explanatory nature, and 6) design. The order of the criteria mentioned was also used to settle ties. All criteria were scored from 1–10 except for design which was scored from 1–5, with 55 being the highest score. The scoring rubric was created on Formstack. Our scoring rubric can be found in Form S5, Supporting Information.

We decided to have each poster judged twice by two different judges and assigned each judge to ten different posters. In order to do this, we contacted early career PIs who had planned on attending the event. To create a fair peer review process, we confirmed that the faculty poster judge was not from the same university as the poster presenter, and also asked this faculty to report any conflict of interests in the posters they were judging. A final count of total judges required was 24. Judges were encouraged to reply to the poster tweets regarding any questions they might have or request additional material (e.g., high resolution poster) they might need to help with their process. A disqualification mechanism was set as well, wherein if the poster was not online by the deadline they would not be judged. It is perhaps prudent to note that a frequent and swift communication is required between judges and organizers as well as organizers and poster presenters to make sure that not only it uses the time available efficiently but also is able to help presenters communicate with judges easily (considering different time zones of either party).

### 9.5 Poster Winner Talk (Day 3) Judging Criteria and Judges

A scoring rubric was created to give talks a quantifiable score which was used to choose two winners. This scoring rubric was similar to the poster judging criteria and included the following categories: 1) research originality, 2) potential impact, 3) comprehensiveness, 4) informative nature, 5) self‐explanatory nature, and 6) presentation skill. Each category was marked from 1–10. The order of the categories mentioned was also used to settle ties. Further details of scoring rubric can be found in Form S6, Supporting Information.

We had requested six attendees to be the judges of student talks. These included editors of Nature Nanotechnology, Nature Materials, C&EN, and ChemSysChem. Also, among the judges were grant program managers from Air Force Office of Scientific Research (US) and John D. and Catherine T. MacArthur Foundation.

## 10 Step 9: Event Execution

### 10.1 Opening the Webinar

Zoom webinar was opened by the *host* 30 min prior to start time, and *co‐hosts* and *panelists* were allowed to join prior to opening the webinar to all Zoom *attendees*. We recommend opening the webinar to the *attendees* at least 5 min before the event start time to allow people to login and join the webinar. On our first day of our event, we experienced a lag when 500+ people were trying to login at the start and received emails from attendees who experienced longer lag times.

### 10.2 Minute Schedule

**Figure 5:**
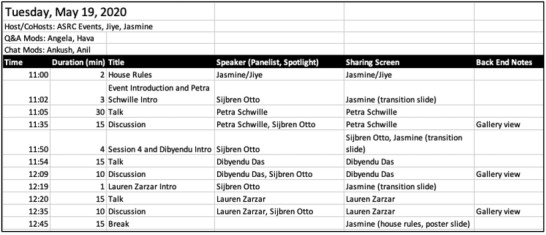
An example of a minute schedule with tasks assigned to two co‐hosts.

A detailed minute schedule prepared in advance is helpful to keep track of the roles for Zoom *co‐hosts*. The schedule was shared between the *co‐hosts* on Google Docs and live edits were made to compensate for delay in schedule. See **Figure** **5** for an example of a minute schedule during a session.

### 10.3 Talk Sessions and Transition Slides

Slides were made for beginning and end of each session and to introduce speakers. House rules were read at the beginning of each session and attendees were encouraged to check out posters, network on Slack, and complete feedback surveys at the end of each session. Slide decks can be found in Form S11, Supporting Information.

### 10.4 Q/A Discussion

*Attendees* had the option to ask questions with mic on or off. By default, all *attendee* mics are disabled on Zoom webinar but can be granted mic access by *co*‐*hosts* when prompted. If the *attendee* did not want to unmute their mic, the session chair would read the question out loud to the speaker. Due to time constraints, unanswered questions were emailed to the speaker and their written reply was posted on the Slack channel with the original question. A sample written replies from Prof. Dibyendu Das to the unanswered questions during Q/A are available in Form S14, Supporting Information.

### 10.5 Slack Networking

A Slack workspace was setup and dedicated channels were made for each session topic, Twitter poster session, and a welcome channel. Attendees were encouraged to introduce themselves on the welcome channel. Information about the Slack channels and the invitation link was displayed prominently on the home page of the event website.^[^ ^4^ ^]^ A sample of the home page with information about Slack is available in Form S15, Supporting Information. Unanswered questions from the event were answered by the speaker, summarized into a PDF, and uploaded onto the respective channel (see Section 11.4).

## 11 Step 10: Post‐Event Analysis and Passing the Baton

### 11.1 Attendance

**Figure 6:**
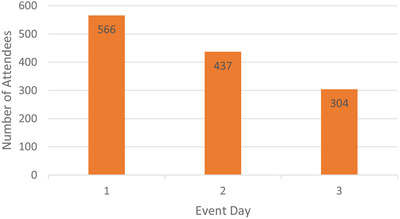
Actual number of attendees on Day 1–3. On‐time registered participants: 977.

We recorded the actual number of attendees on Day 1 through 3. The highest turnout rate was on Day 1 (58%) with decreasing turnout rates on day 2 (45%) and day 3 (31%) (**Figure** **6**).

### 11.2 Feedback Survey

An anonymous feedback survey was created and announced during the end of sessions on Day 3. When the event concluded, a thank you email was sent to all the registered attendees and included the feedback survey. Event feedback form can be found in Form S9, Supporting Information

**Figure 7:**
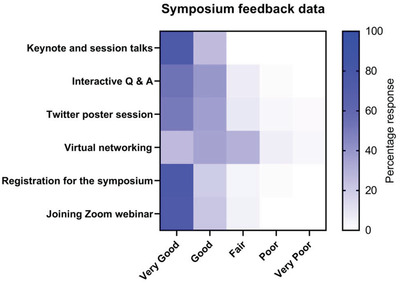
Symposium feedback data from 103 anonymous participant surveys.

From the survey feedback (**Figure** **7**), the participants found the talk sessions, registration process, and joining the webinar on Zoom to be mainly a *very good* experience. The interactive Q/A and the Twitter poster session were reported to be a *very good* to a *good* experience. Virtual networking was rated the lowest, as a *good* to a *fair* experience.

Poster and networking sessions on virtual platforms are most difficult due to the unfamiliarity of the experience. One software program called Gather.town tries to emulate the in‐person poster session by having an avatar walk around and approach the avatar of a poster presenter. Upon two or more avatars being gathered together, the people in the group will be joined by a video call. Gather.town was used by the 2021 System Chemistry Symposium's poster session which allowed for real‐time discussions and networking (Section 11.5). While some people prefer Gather.town over Twitter for the real‐time discussions, others noted that Gather.town is more socially awkward to use because they felt that abruptly joining a group of people in an on‐going conversation is intrusive without having the body‐language cues to know when it is appropriate to jump in the conversation.

We will continue to experiment with different software programs or methods to increase the level of engagement in virtual poster and networking sessions.

### 11.3 Certificate of Attendance

Certificates of attendance were created for those who requested one. An example of a certificate of attendance can be found in Form S10, Supporting Information.

### 11.4 Twitter Metrics

After the event we asked the presenters to email us their tweet analytics as they stood at the end of the symposium. Of the total number of posters, around 30% participants mailed us back with the details. We averaged the impression and engagements (4046 and 605, respectively) and extrapolated it over 105 participants.

### 11.5 Passing the Baton

If an event is particularly successful, there may be interest to host a follow‐up. In the case of System Chemistry, another virtual event was held in 2021 and another is planned for 2022. All materials were passed on to the subsequent organizers and they hosted an excellent virtual symposium with improved changes. For example, SysChem21 used Gather Town to create a virtually interactive and engaging poster session which led to more emphasis on joint discussions after talks.

## 12 Conclusion

In conclusion, it took 2 months of intense planning and a total of 66 people involved to reimagine a symposium in the virtual format that was scientifically stimulating, community engaging, and enjoyable. It is extremely rewarding as organizers to receive positive and constructive feedback from the community on their event experience. We hope that these guidelines and resources will be shared widely and help scientists to continue sharing and communicating their research with the virtual, global community.

## Conflict of Interest

The authors declare no conflict of interest.

## Supporting information

Supporting Information
