# Supplementary material for: Ten Steps to Organize a Virtual Scientific Symposium and Engage Your Global Audience
Source: Glob Chall. 2022 Jun 26;6(9):2200005. doi: 10.1002/gch2.202200005 (PMC9463518; doi:10.1002/gch2.202200005)
Supplement: Supplementary file 1 — Supporting Information [file GCH2-6-2200005-s001.pdf]

Global Challenges
Open Access

Supporting Information

for *Global Challenges,* DOI: 10.1002/gch2.202200005

Ten Steps to Organize a Virtual Scientific Symposium and Engage Your Global Audience

Jiye Son, Jasmine Sabio, Ankit Jain, and Rein V. Ulijn**

Supporting Information

Ten Steps to Organize a Virtual Scientific Symposium and Engage Your Global Audience

Jiye Son, Jasmine Sabio, Ankit Jain, and Rein V. Ulijn**

The following forms are enclosed in this Supporting Information document:

S1. Speaker guide
S2. Session Chair guide
S3. Q&A and Chat Moderator guide
S4. Participant guide
S5. Twitter Poster scoring rubric
S6. Poster Winner Talk scoring rubric
S7. Event registration form
S8. Poster registration form
S9. Event feedback form
S10. Certificate of attendance
S11. Event program – Sample of Day 1
S12. Twitter Presentation session – Sample of a session with presenter list
S13. Information for Twitter Poster presenters – Sample of resources
S14. Written responses to unanswered questions – Sample of Prof. Dibyendu Das
S15. Event website – Sample of home page with information on Slack

The following slide deck is available as a separate PowerPoint file in Supporting Information:

S16. Son_Virtual_Talk Session Transition Slides.pptx

VIRTUAL SYMPOSIUM

SYSTEMS CHEMISTRY

Life-like emergent behavior in complex molecules and ensembles

Interactive Talks, Keynotes & an Interactive Twitter-Based Poster Session

@syschem20

8 AM PDT | 11 AM EDT | 3 PM GMT | 5 PM CET

MAY 18-20, 2020

Ben-Gurion University of the Negev

EMORY UNIVERSITY

מכון ויצמן למדע
WEIZMANN INSTITUTE OF SCIENCE

university of groningen

JOHNS HOPKINS UNIVERSITY

ADVANCED SCIENCE RESEARCH CENTER
THE GRADUATE CENTER
CITY UNIVERSITY OF NEW YORK

Speakers as panelists

- Join at least 30 minutes before your talk
- Will join as “attendees” on the day of the event
- We will change your status to “panelist” before your talk (as the speaker before you is finishing up Q&A)
- After your talk is over, we will change you back to “attendees”

- Panelists → video, microphone, share screen, chat, CAN’T ask questions
- Attendees → chat, ask questions, mic on when granted

Speaker presentation: introduction

- Your session chair will introduce you

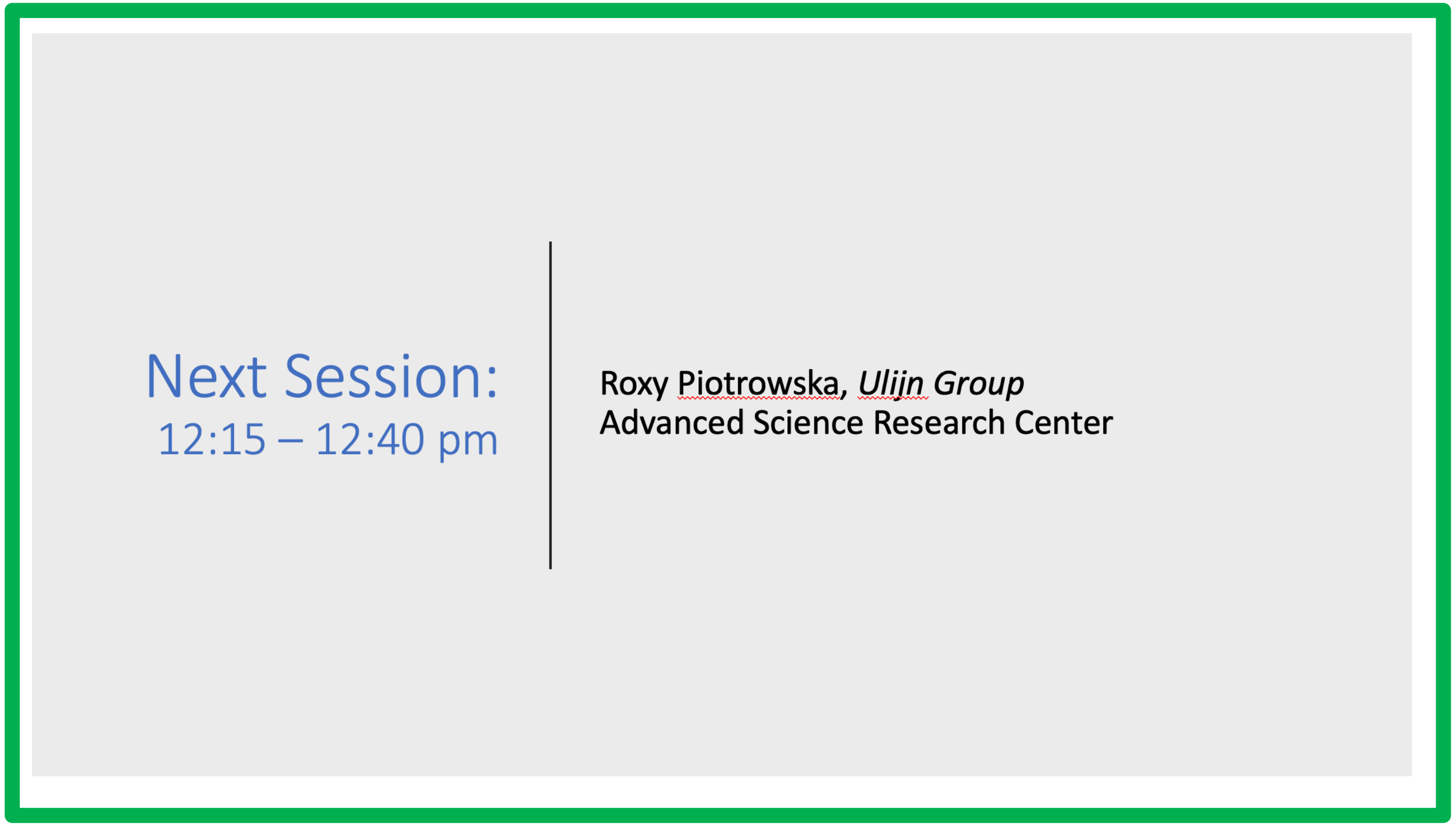

Speaker presentation: sharing screen

- Share your Power Point, etc., not your Desktop
- Close additional programs, don't download/upload files
- Turn off your email notifications!
- Check connection: https://fast.com (2-6 Mbps needed for Zoom)

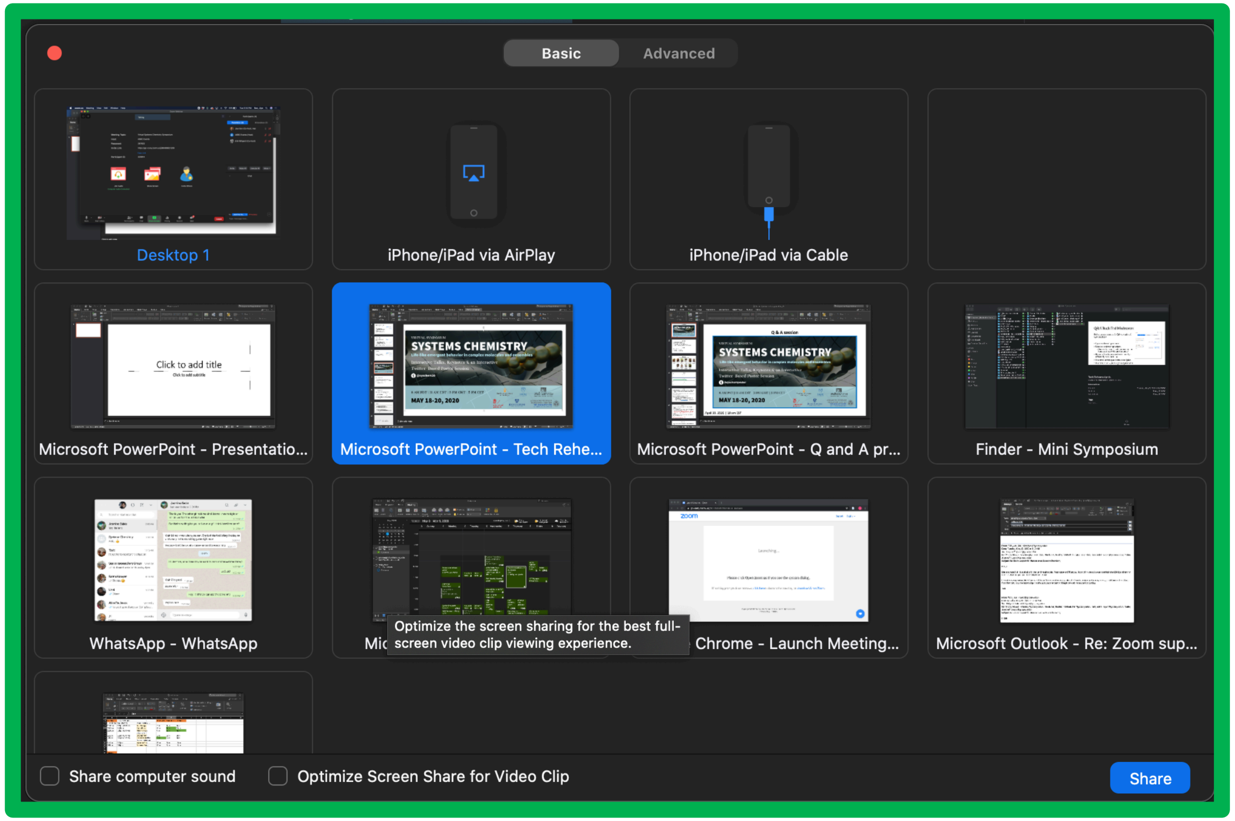

Speaker presentation: during presentation

- We will highlight your video
- Jiye will give 5 minute warning

Speaker presentation: Q&A

- Your session chair will moderate the Q&A box:
    A) Session chair will read out loud the question
    B) We will allow attendees to unmute their microphone and ask you the question directly

Speaker presentation: after presentation

- Turn off your video and microphone
- We will change your status to “attendees”
- All unanswered questions will be emailed to you if you’d like to follow up with the person
- Enjoy the symposium!

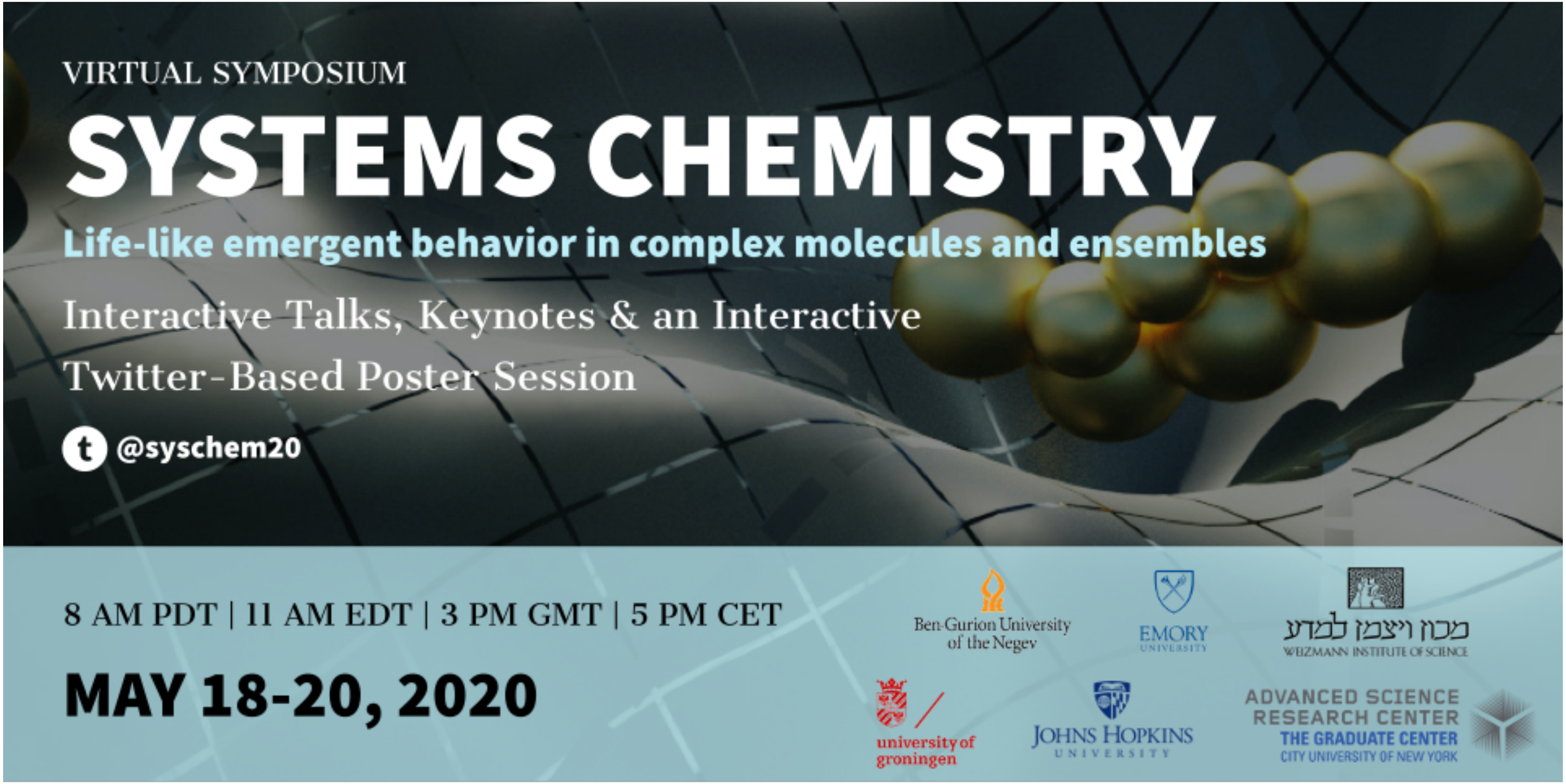
VIRTUAL SYMPOSIUM
SYSTEMS CHEMISTRY
Life-like emergent behavior in complex molecules and ensembles
Interactive Talks, Keynotes & an Interactive Twitter-Based Poster Session
@syschem20
8 AM PDT | 11 AM EDT | 3 PM GMT | 5 PM CET
MAY 18-20, 2020
Ben-Gurion University of the Negev
EMORY UNIVERSITY
מכון ויצמן למדע
WEIZMANN INSTITUTE OF SCIENCE
university of groningen
JOHNS HOPKINS UNIVERSITY
ADVANCED SCIENCE RESEARCH CENTER
THE GRADUATE CENTER
CITY UNIVERSITY OF NEW YORK

Session Chair Guide

Systems Chemistry Virtual Symposium 2020

Example Session Breakdown

Time (EDT)	Duration (min)	Title	Speaker	Sharing Screen
12:00	4	Session 1 and Sarah Perry Intro	Gonen Ashkenasy	Gonen Ashkenasy
12:04	15	Talk	Sarah Perry	Sarah Perry
12:19	10	Discussion	Sarah Perry, Gonen Ashkenasy	Sarah Perry
12:29	1	Peter Korevaar Intro	Gonen Ashkenasy	Jasmine (transition slide)
12:30	15	Talk	Peter Korevaar	Peter Korevaar
12:45	10	Discussion	Peter Korevaar, Gonen Ashkenasy	Peter Korevaar
12:55	15	Break		Jasmine (house rules, poster slide)

Housekeeping

- Mic off when you’re not speaking
- Turn off computer notifications (emails, messaging apps, etc.)
- Check connection: https://fast.com (2-6 Mbps needed for Zoom)
  - Thanks Rafal for the tip!

Session/First Speaker Introduction - 4 min

- Mic on, Video on
- Introduce the session topic
  - Feel free to use slides
- Introduce the first speaker in your session
  - Jasmine will share her screen to provide a title slide

Second Speaker Introduction - 1 min

- Mic on, video on
- Introduce the second speaker in your session
  - Jasmine will share her screen to provide a title slide

Q&A Session

- Mic on, video on
- Have Q&A window open with the "Open" tab[1]
- Read top question in the open question queue[2]
  - Moderators will dismiss questions for you as they are answered
- Introduce the participant name and their institution before reading the question
- If participant wants to ask their question, give them ~10 seconds to ask, if silent, go ahead and read the question
- Be mindful of time

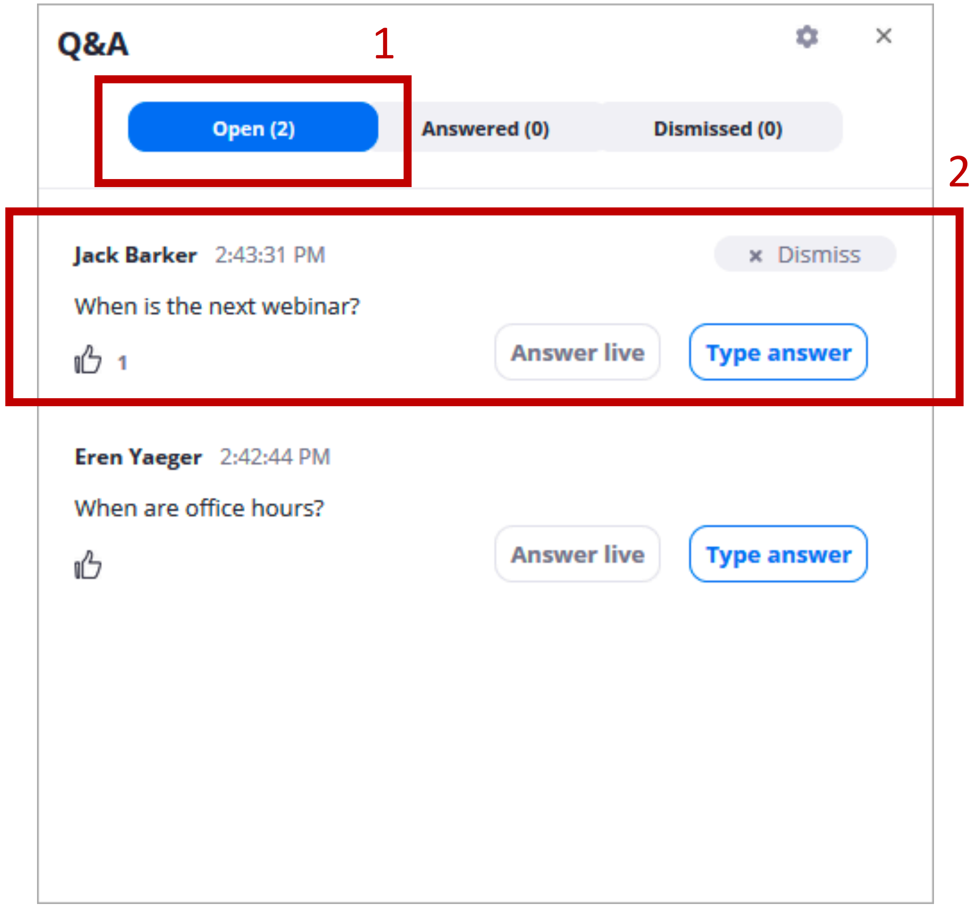

Q&A Back End Moderators Role

Role: queue questions in Q&A window for session chairs

- Dismiss irrelevant questions
  - Dismissed questions can be undismissed and transferred back to the question queue*
- Be on call with your moderator buddy to efficiently sort questions
- You can't submit questions to the Q&A
- You'll be down casted to participant when you're not an active moderator

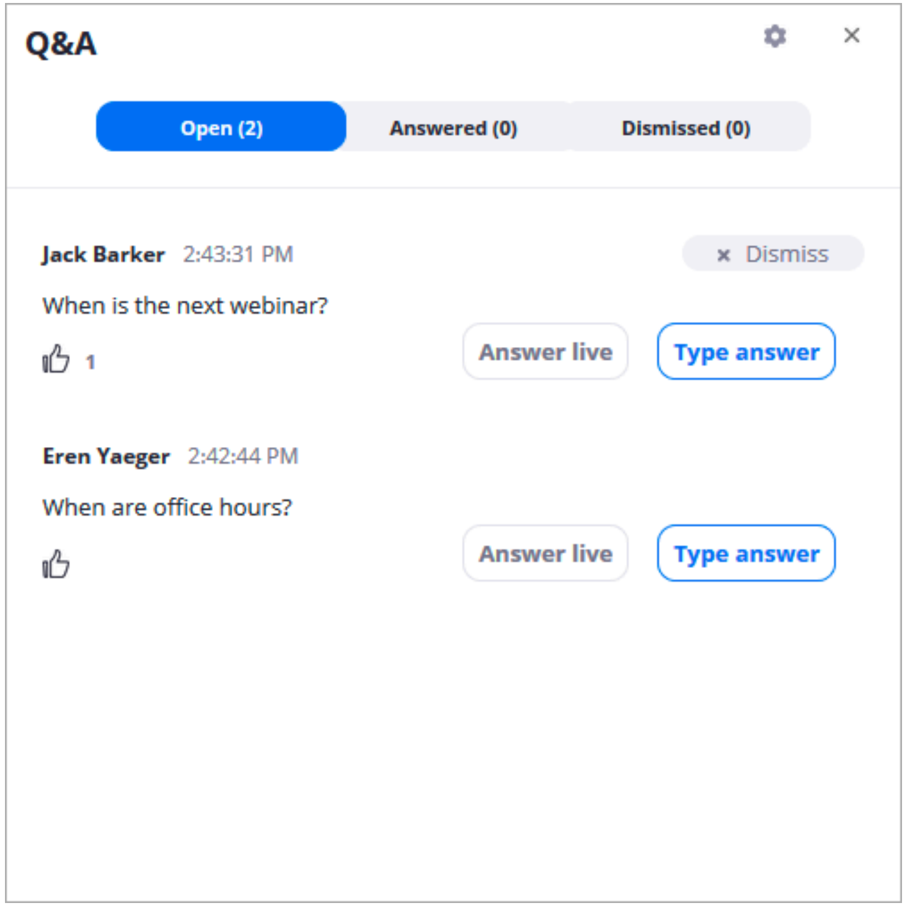

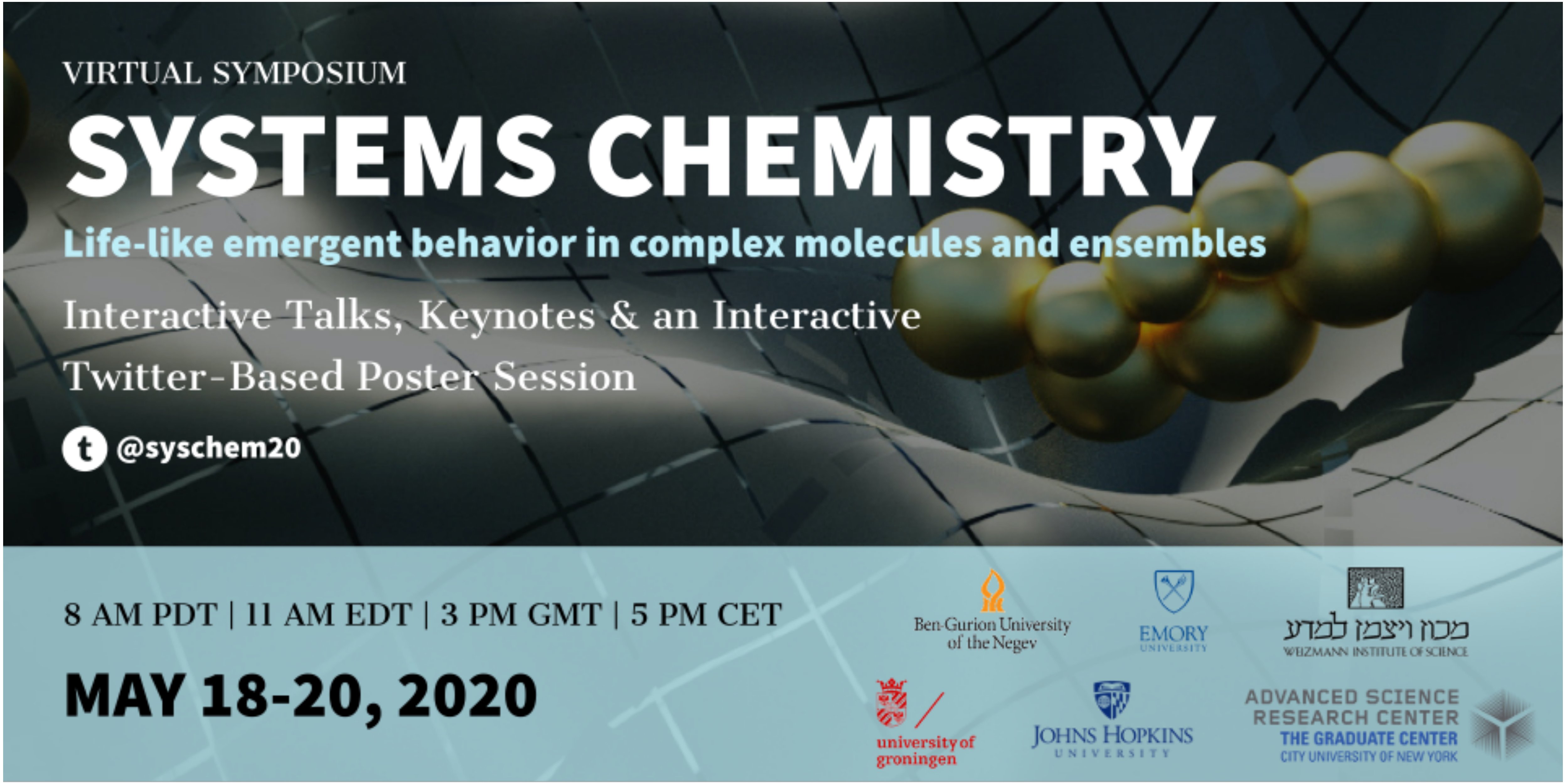
VIRTUAL SYMPOSIUM
SYSTEMS CHEMISTRY
Life-like emergent behavior in complex molecules and ensembles
Interactive Talks, Keynotes & an Interactive
Twitter-Based Poster Session
@syschem20
8 AM PDT | 11 AM EDT | 3 PM GMT | 5 PM CET
MAY 18-20, 2020
Ben-Gurion University of the Negev
EMORY UNIVERSITY
מכון ויצמן למדע
WEIZMANN INSTITUTE OF SCIENCE
university of groningen
JOHNS HOPKINS UNIVERSITY
ADVANCED SCIENCE RESEARCH CENTER
THE GRADUATE CENTER
CITY UNIVERSITY OF NEW YORK

Moderator Guide

Systems Chemistry Virtual Symposium 2020

Q&A Back End Moderators - Role

Role: dismiss questions in Q&A window for session chairs

- Video off, mic off
- You can’t submit questions to the Q&A
- You’ll be down casted to participant when you’re not an active moderator

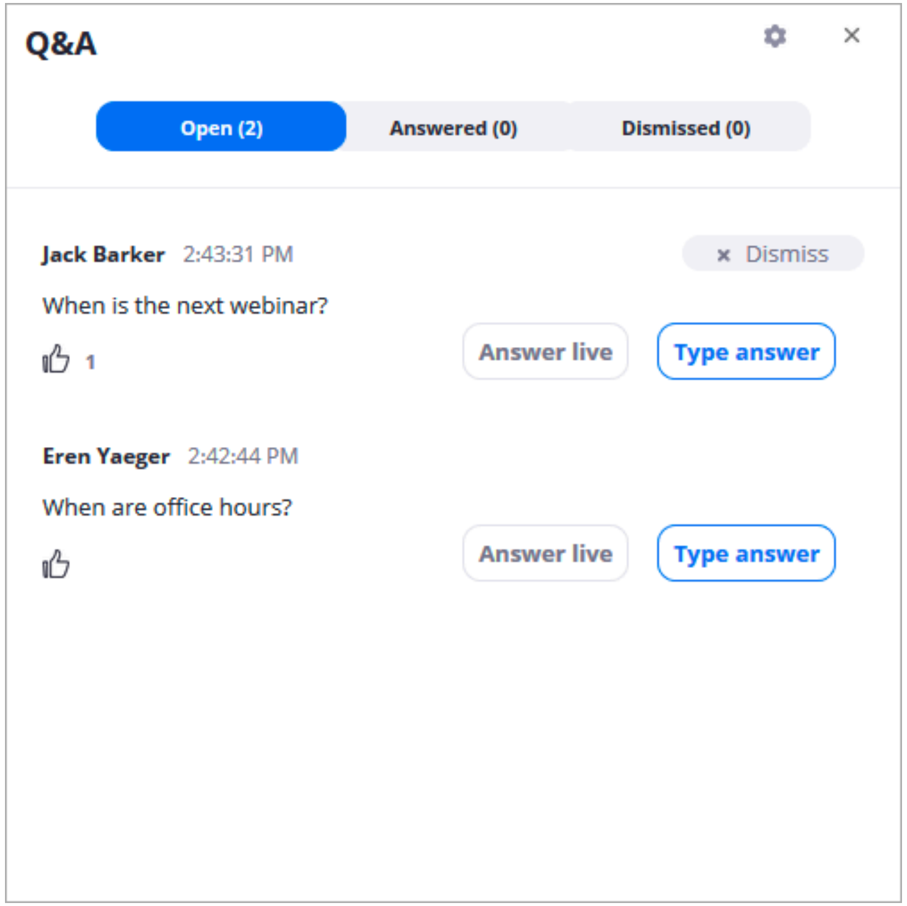

Q&A Back End Moderators - Instructions

- Dismiss irrelevant questions
  - Dismissed questions can be undismissed and transferred back to the question queue
- When session chair asks a question, press ‘Answer live’ for that question and press ‘Done’ when question has been answered
  - We have instructed session chairs to read the top questions but they’re free to ask any question
- Be on call with your moderator buddy to efficiently sort questions

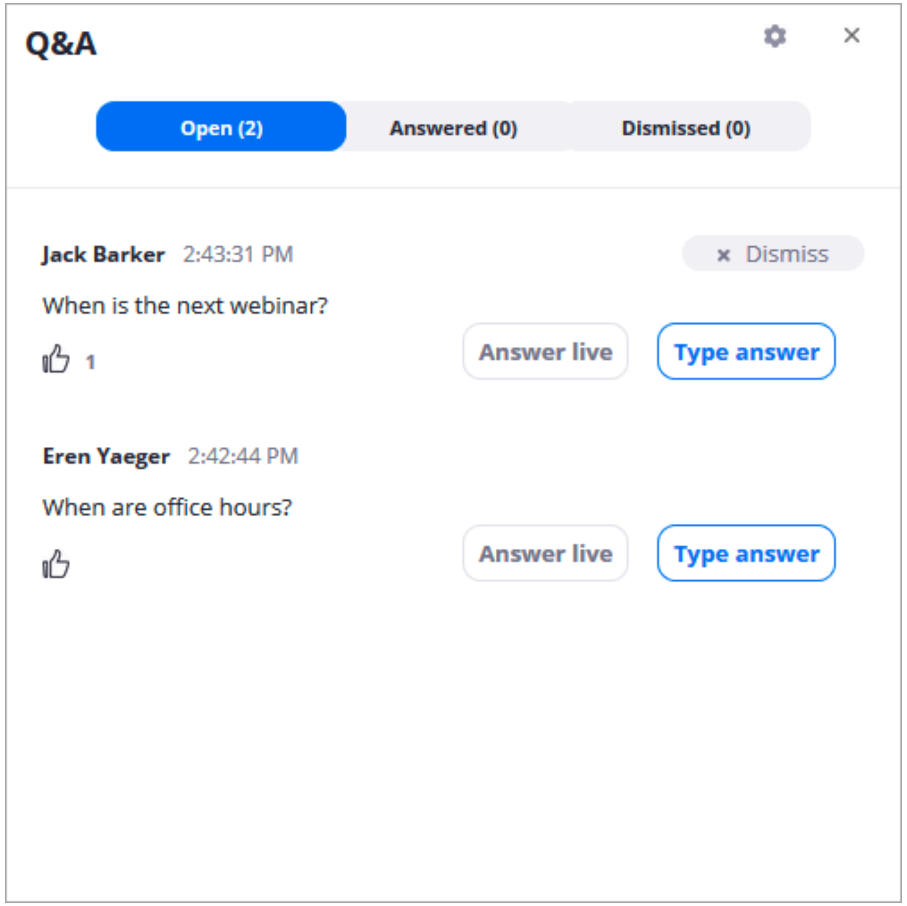

Chat Moderators - Role

Role: moderate chat and insert breaks into the Q&A

- You won’t have mic/video access
- Report inappropriate attendees to Jiye or Jasmine

At the end of a discussion, submit “THIS CONCLUDES *insert speaker’s name* DISCUSSION. ALL FOLLOWING QUESTIONS ARE DIRECTED FOR *insert next speaker’s name* DISCUSSION.

VIRTUAL SYMPOSIUM

SYSTEMS CHEMISTRY

Life-like emergent behavior in complex molecules and ensembles

Interactive Talks, Keynotes & an Interactive Twitter-Based Poster Session

@syschem20

8 AM PDT | 11 AM EDT | 3 PM GMT | 5 PM CET

MAY 18-20, 2020

Ben-Gurion University of the Negev

EMORY UNIVERSITY

מכון ויצמן למדע
WEIZMANN INSTITUTE OF SCIENCE

university of groningen

JOHNS HOPKINS UNIVERSITY

ADVANCED SCIENCE RESEARCH CENTER
THE GRADUATE CENTER
CITY UNIVERSITY OF NEW YORK

Participant Guide

Systems Chemistry Virtual Symposium 2020

Rules of Conduct

- Organizers will not tolerate inappropriate behavior and online harassment of any kind.
- Comments that demean another person due to his or her gender, gender identity or expression, race, religion, ethnicity, age or disability will not be tolerated.
- Review of such allegations will be conducted on a case by case basis. Perpetrators can face temporary/permanent ban from the online symposium.
- Organizers always expect the participants to abide by this code during the symposium and Twitter poster event. Failure in doing so shall result in a void registration and additional appropriate action.

Zoom Webinar - Instructions

- Install the latest version of Zoom Desktop Client for full functionality of the webinar
- Join the webinar with the invitation link or manually entering the meeting ID into Zoom
  - You do not need to set up a zoom account to participate in the webinar
  - Enter your first and last name into the 'Your Name' field

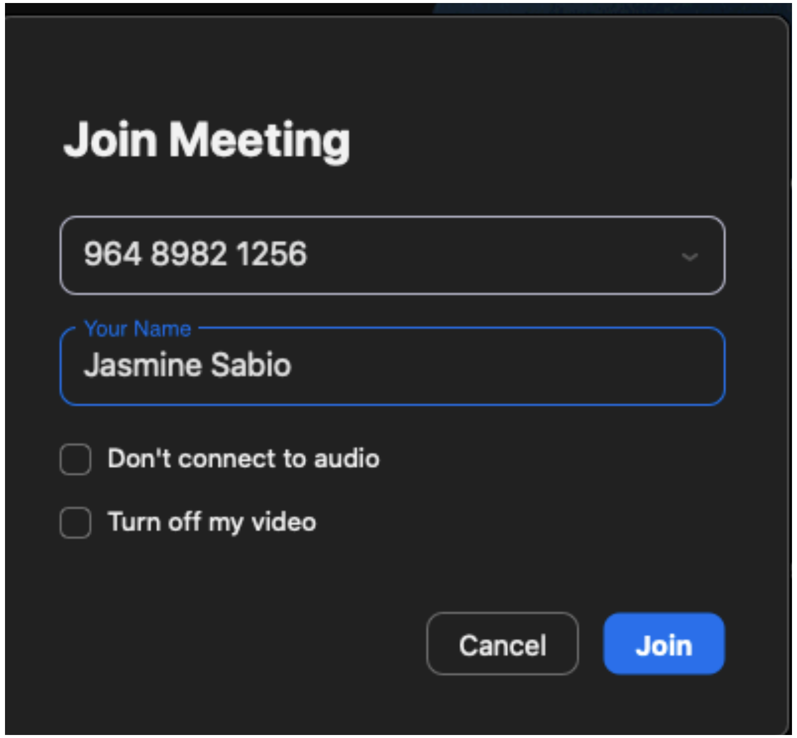

Zoom Documentation for Joining and Participating in a Webinar

Zoom Webinar – View Settings

- Window and full screen options available

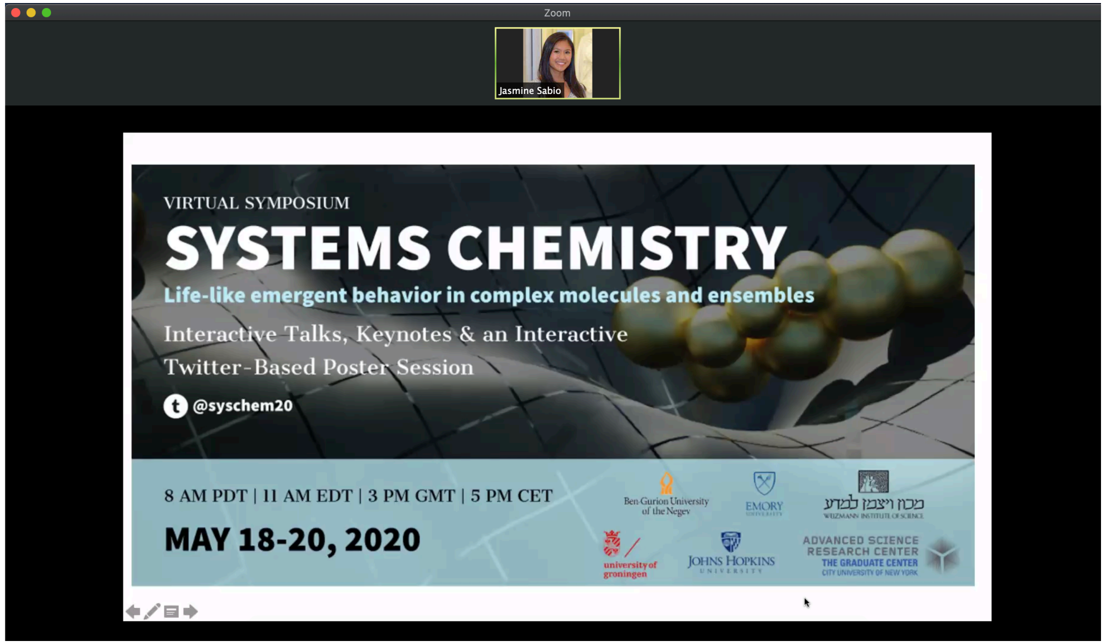

Window Screen

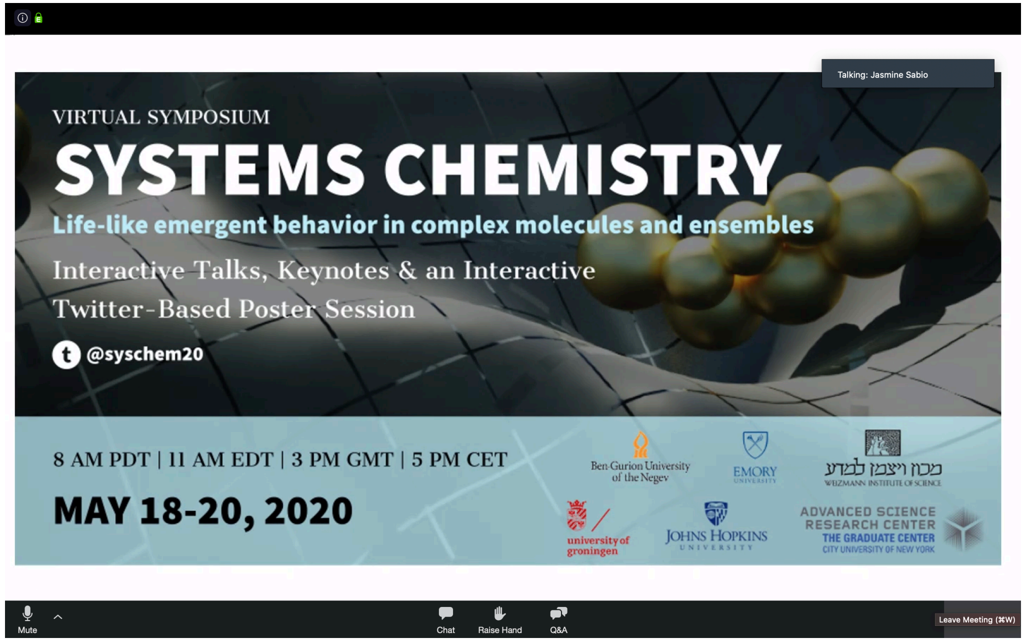

Full Screen

Zoom Webinar – View Settings

- Toggle between views in top right corner of Zoom window

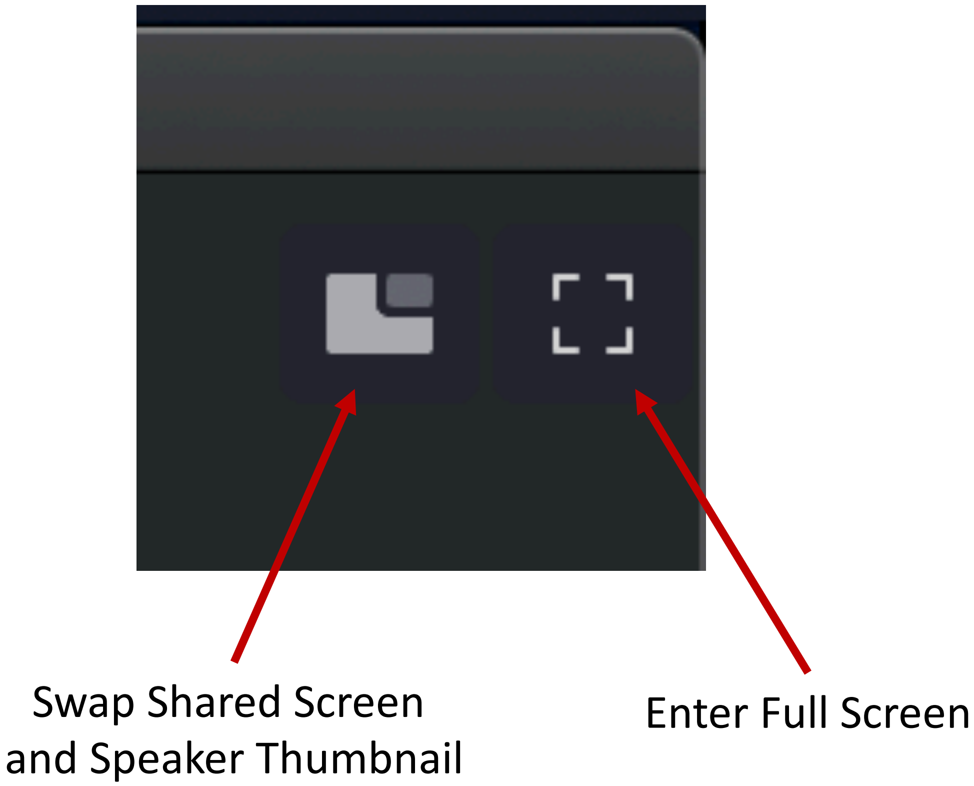

Zoom Webinar - View Settings: Full Screen

- Toggle between show active speaker video or hide thumbnail video
- Move thumbnail video by clicking and dragging across the screen
- Resize thumbnail video by expanding the bottom corners

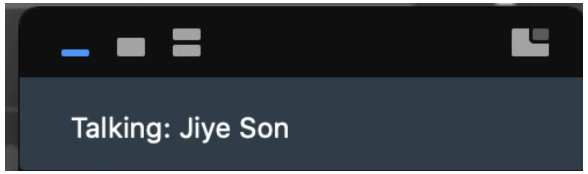

View Controls appear when you hover over the thumbnail

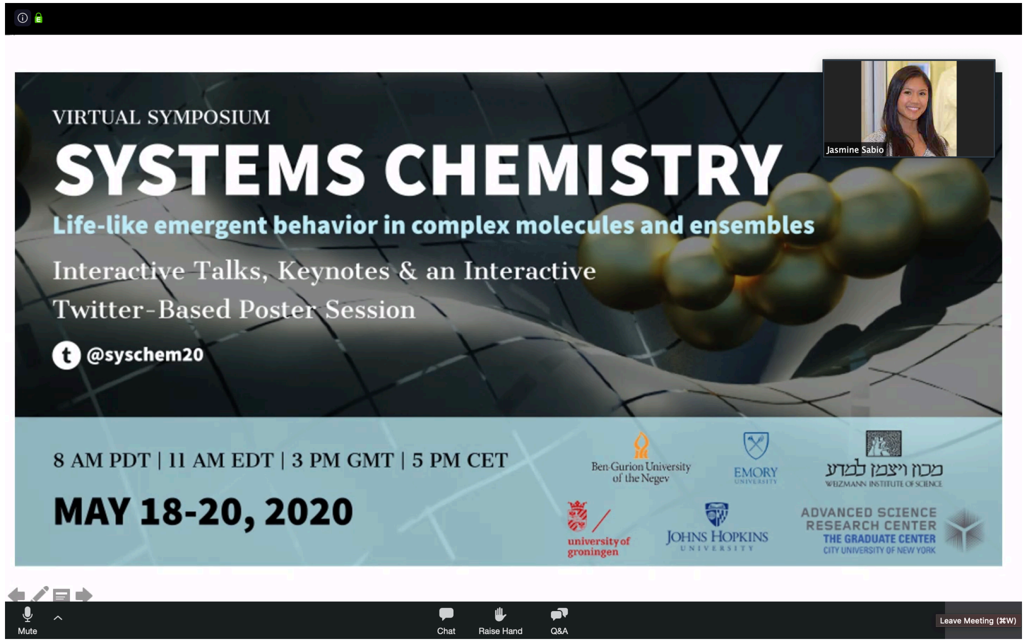

Show Active Speaker Video

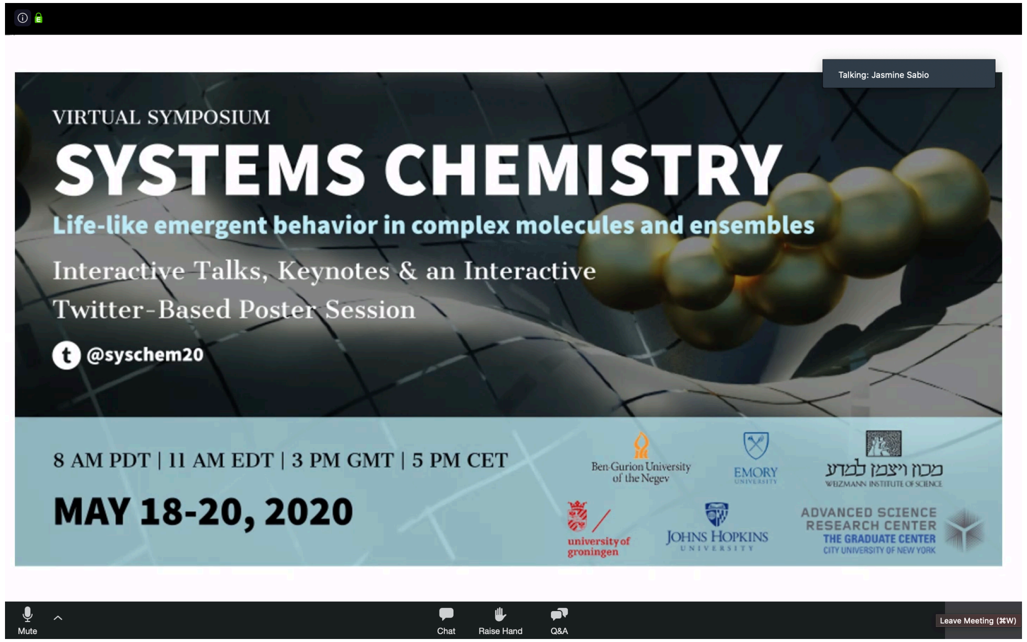

Hide Thumbnail Video

Zoom Webinar - Chat

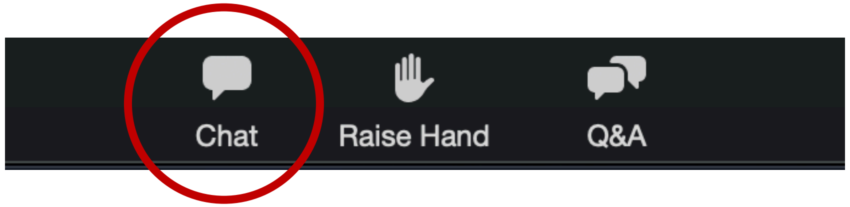

- Chat directly to panelists or to everyone in the chat box
  - Toggle between ‘All panelists’ and ‘All panelists and attendees’
- Feel free to make comments throughout the event!

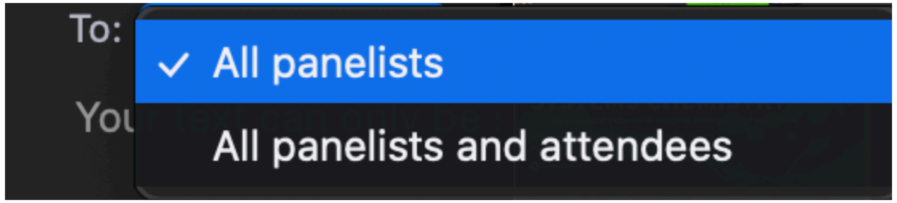

Zoom Webinar – Q&A

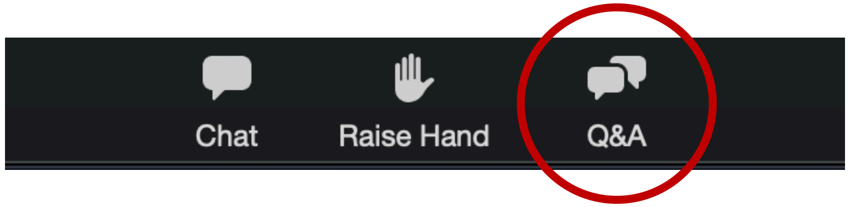

- Pose questions in the Q&A box
- You will have the option to ask your question live with your mic on – it must be indicated in your question
- Due to time and the amount of participants, we may not get through all the questions
  - We will follow up offline for all unanswered questions

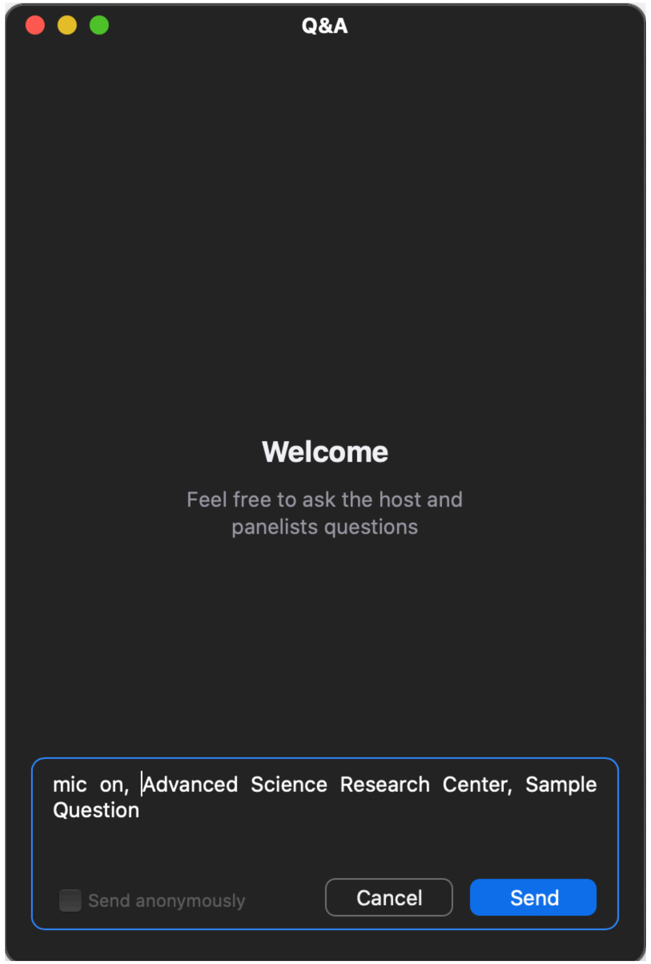

Question format: mic on/off, institution, question

Slack - Instructions

- Keep the discussion going on Slack! Chat about the symposium, Twitter poster session, or meet other attendees
- Can be accessed through browser or desktop/mobile app

Join with this invite link.

Slack – Join Channels

- You'll automatically be joined to the #welcome channel
  - Feel free to introduce yourself!
- Join channels for the different sessions
  - #dynamic: Dynamic Information of Molecular Assemblies
  - #origins: Origins and Synthesis of Life
  - #emergent: Emergent Behaviors: From Catalysts to Motility
  - #active: Active and Adaptive Materials
  - #bionet: Biological Networks
  - #covid-19: Systems Chemistry and the Coronavirus Crisis
  - #posters: Twitter Poster Session
  - #random: random things you want to talk about!
  - #questions: open questions you have for the organizers

Slack – Join Channels

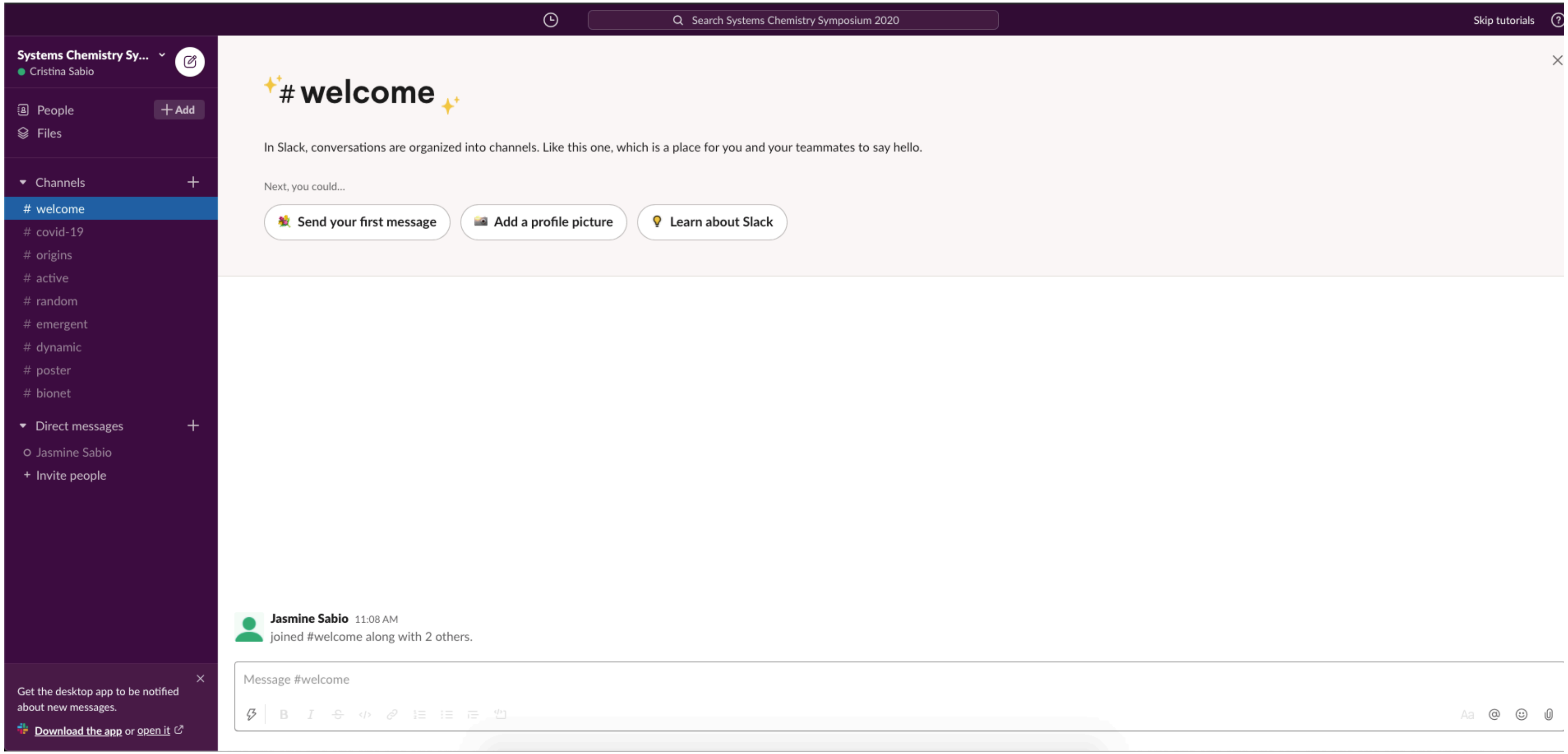

Join Channels

Poster Evaluation

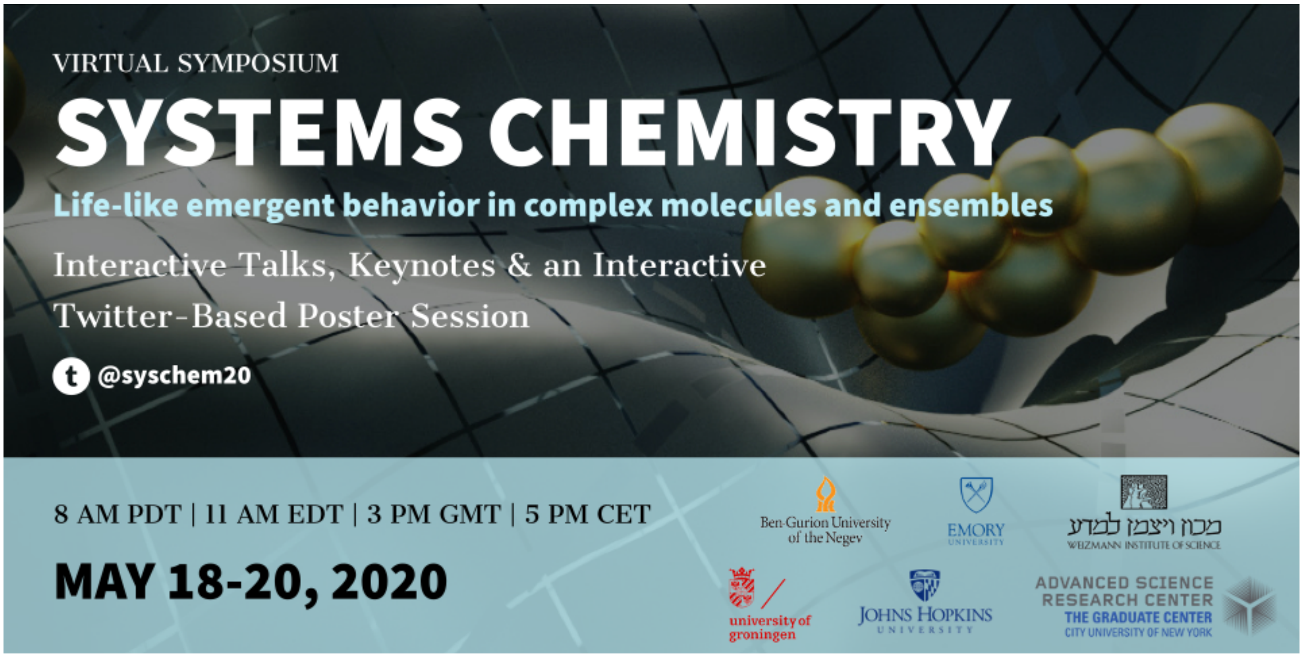

Please submit all poster evaluations by **Tuesday, May 19, at 11:00 am EDT**.

Evaluation Criteria

- Research Originality: novelty of the work

- Potential Impact: broader scientific or societal impact of the research

- Comprehensiveness: exhaustive nature and cohesiveness of the study presented

- Informative: poster information and supporting information is descriptive and educational

- Self-Explanatory: poster can be understood by any scientific audience and is understood sufficiently on its own

- Design: do the aesthetics of the poster clearly support the research

Your Name *

First Name

Powered by Formstack Create your own form ›

Poster Presenter Name *

Poster Subcategory *

- #dynamic
- #origins
- #emergent
- #active
- #bionet

Poster Evaluation *

	1 - Do not agree	2	3	4	5	6	7	8	9	10 - Highly agree
Research Originality	○	○	○	○	○	○	○	○	○	○
Potential Impact	○	○	○	○	○	○	○	○	○	○
Comprehensiveness	○	○	○	○	○	○	○	○	○	○
Informative	○	○	○	○	○	○	○	○	○	○
Self-Explanatory	○	○	○	○	○	○	○	○	○	○
Design	○	○	○	○	○	○	○	○	○	○

Additional Comments

If presenter is chosen to present their work, we can relay any specific comments to them.

Powered by Formstack Create your own form ›

Submit Form

Powered by Formstack Create your own form ›

Presentation Evaluation

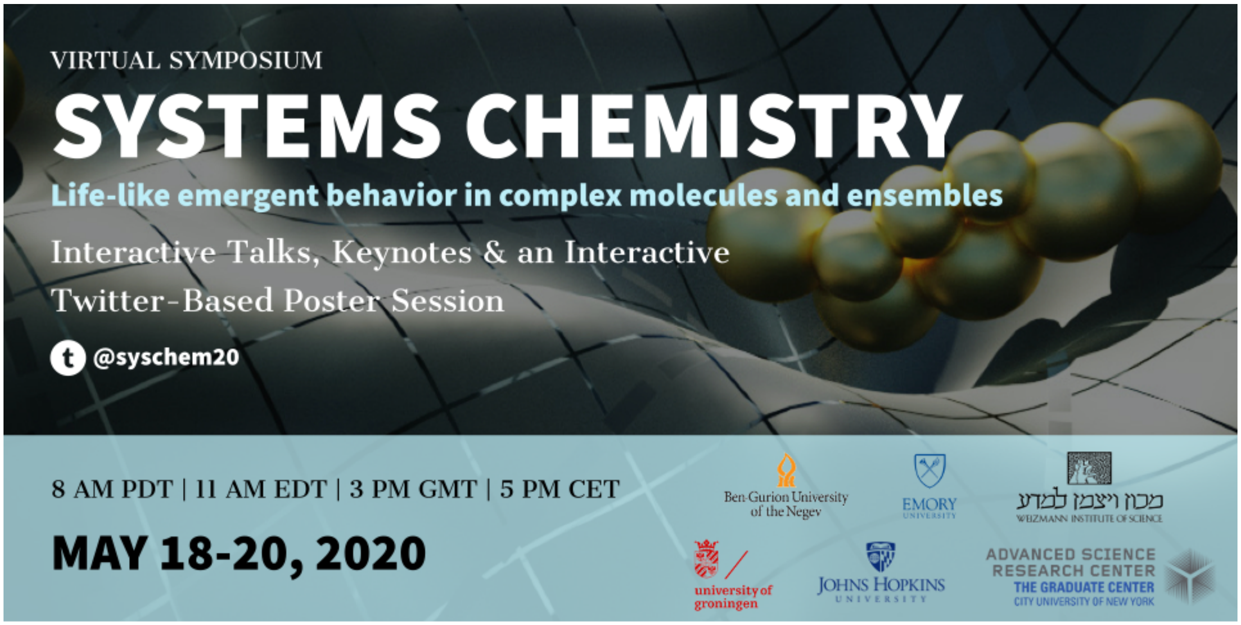

Evaluation Criteria

Research Originality: novelty of the work

Potential Impact: broader scientific or societal impact of the research

Comprehensiveness: exhaustive nature and cohesiveness of the study presented

Informative: descriptive and educational nature of the presentation

Presentation Skill: clarity of slides and the speaker

Judge's Name *

First Name

Last Name

Name of Presenter *

Powered by Formstack Create your own form ›

	1 - Do not agree	2	3	4	5	6	7	8	9	10 - Highly agree
Research Originality	○	○	○	○	○	○	○	○	○	○
Potential Impact	○	○	○	○	○	○	○	○	○	○
Comprehensiveness	○	○	○	○	○	○	○	○	○	○
Informative	○	○	○	○	○	○	○	○	○	○
Presentation Skill	○	○	○	○	○	○	○	○	○	○

Additional Comments

Submit Form

Powered by Formstack Create your own form ›

Event Registration

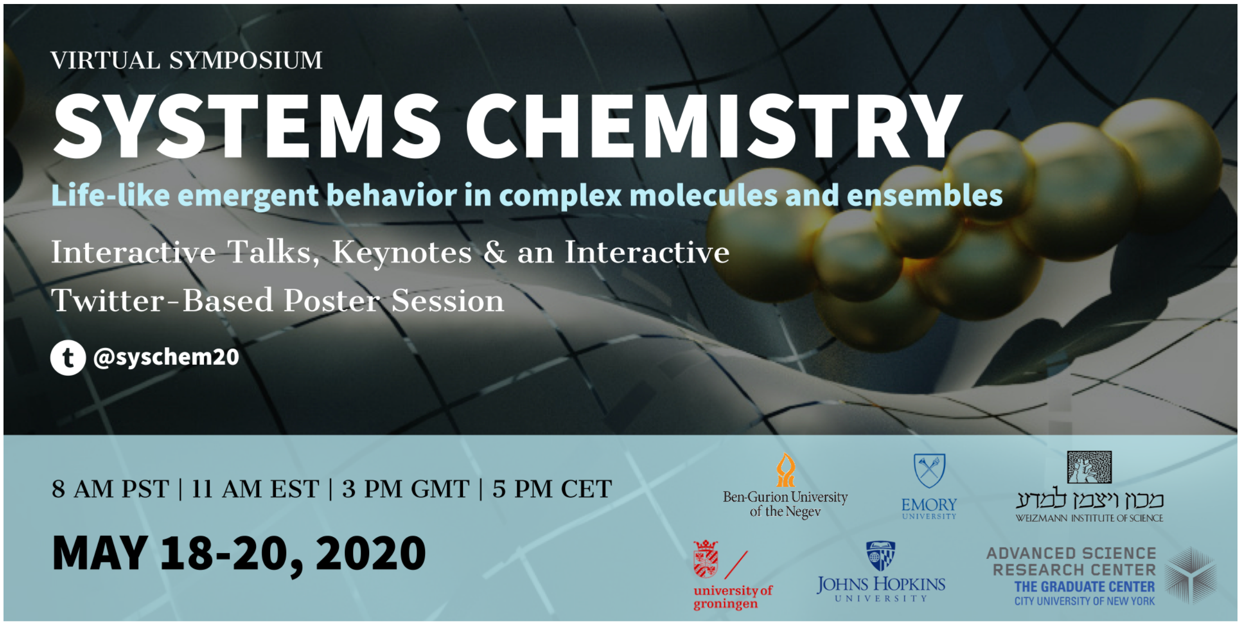

Please fill out your information below to register for this event. Zoom access instructions emailed to the email address provided. Registration closes on **May 4, 2020 at 12 pm EST**.

Name *

First Name

Last Name

Email *

Title (PhD Student, Post-Doc, Professor, etc.) *

Lab Group *

Powered by Formstack Create your own form ›

Department *

Institution/Organization *

Location (City, Country) *

What research topics are you most interested in? *

- ☐ Dynamic Information of Molecular Assemblies
- ☐ Origins and Synthesis of Life
- ☐ Emergent Behaviors: From Catalysts to Motility
- ☐ Active and Adaptive Materials
- ☐ Biological Networks, from Tissues to Oceans
- ☐ Systems Chemistry and the Coronavirus Crisis

Check all the apply.

Will you present a poster? *

- ○ Yes
- ○ No

Would you like to sign up to receive updates and event notifications from the Nanoscience Initiative at the Advanced Science Research Center? *

- ○ Yes
- ○ No

Powered by Formstack Create your own form ›

Submit Form

Powered by Formstack Create your own form ›

Poster Session Survey

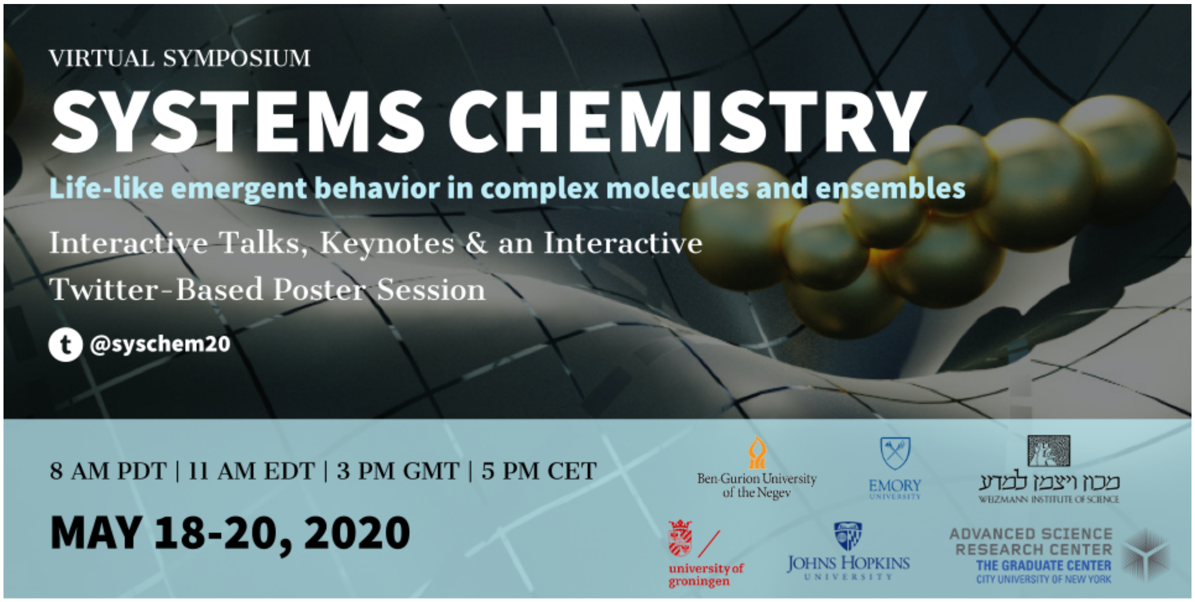

Survey is required and must be completed **by May 10 at 12:00 PM EDT** to participate in the Systems Chemistry poster session.

Name *

First Name

Last Name

Email *

Twitter Handle *

Twitter handles appear after the @ sign in your profile URL and it must be unique to your account e.g. @syschem20, @asrc_gc

Poster Title *

Powered by Formstack Create your own form ›

Designated Poster Sub-Category

- #dynamic
- #origins
- #emergent
- #active
- #bionet

If selected, I will present my poster topic with a 15 minute talk on May 20 for a chance to win the poster prize. *

- Yes
- No

Submit Form

Powered by Formstack Create your own form ›

Event Feedback Form

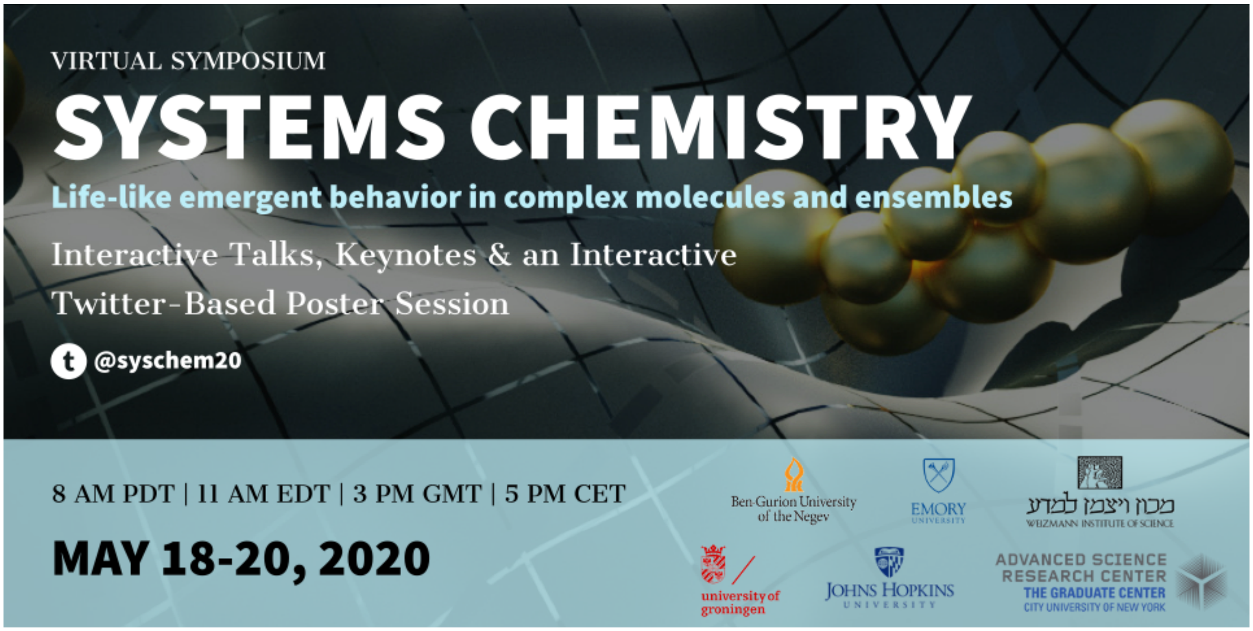

I am a *

- Undergraduate Student
- Graduate Student
- Postdoc
- Early Career Faculty
- Tenured Faculty

I identify as *

- Female
- Male
- Other

Powered by Formstack Create your own form ›

◯ Prefer not to say

How would you rate this virtual experience?

	Very Good	Good	Fair	Poor	Very Poor
Keynotes and session talks	◯	◯	◯	◯	◯
Interactive Q&A session	◯	◯	◯	◯	◯
Twitter poster session	◯	◯	◯	◯	◯
Virtual networking	◯	◯	◯	◯	◯
Registering for the symposium	◯	◯	◯	◯	◯
Joining the Zoom webinar	◯	◯	◯	◯	◯

How did you hear about the symposium? *

Do you have comments or feedback for the organizers?

Submit Form

Powered by Formstack Create your own form ›

(University Heading)

June 9, 2020

Re: Certificate of Attendance for XXX

To Whom It May Concern:

This letter is to certify that XXX attended the **Virtual Systems Chemistry Symposium** held from May 18, 2020 to May 20, 2020 and presented a poster during the Virtual Twitter Poster Session.

More information on the symposium can be found on:
https://asrc.gc.cuny.edu/nanoscience/events/virtual-symposium-systems-chemistry/

Sincerely,
XXX

XXX
Chair of the Virtual Systems Chemistry Symposium

NANOSCIENCE INITIATIVE
ADVANCED SCIENCE RESEARCH CENTER

Program

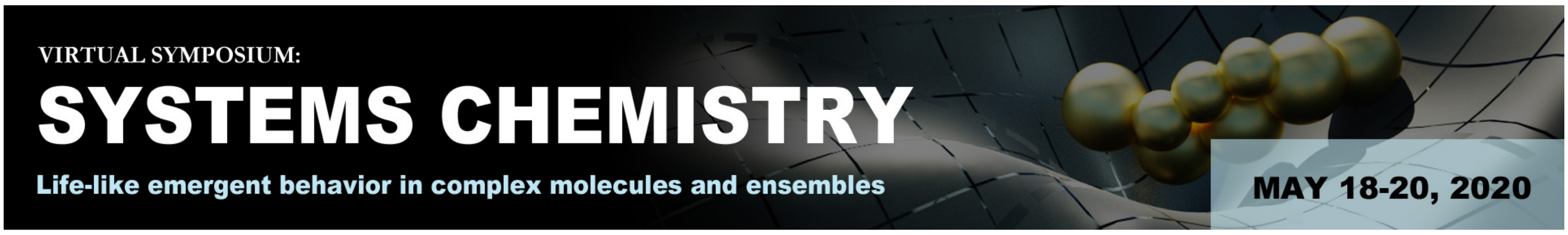

[Home](#)
[Speakers](#)
[Program](#)
[Twitter Poster Session](#)
[Information for Presenters](#)
[Organizers](#)

Follow the Event @syschem20

Contact Us

nanoscience@gc.cuny.edu

Program

All times listed are Eastern Daylight Time (EDT). All sessions will be hosted on Zoom.

Check out our Participant Guide (PDF) for a summary of our Rules of Conduct, Zoom instructions, and Slack instructions.

– Monday, May 18, 2020

11:00 a.m.	Opening Remarks<br>**Meeting Organizers**
11:15 a.m.	KEYNOTE: Dynamic molecular systems<br>**Ben Feringa**, *University of Groningen*
12:00 p.m.	SESSION 1: Dynamic Information of Molecular Assemblies<br>**Chair: Gonen Ashkenasy**, *Ben-Gurion University of the Negev*
12:05 p.m.	Designing Phase Separation in Complex Systems (PDF)<br>**Sarah Perry**, *University of Massachusetts Amherst*
12:30 p.m.	Programmable life-like materials that organize at mesoscopic scale (PDF)<br>**Peter Korevaar**, *Radboud University Nijmegen*
12:55 p.m.	Break
1:10 p.m.	SESSION 2: Origins and Synthesis of Life<br>**Chair: David Lynn**, *Emory University*
1:15 p.m.	The systems chemistry of life-like objects (PDF)<br>**Stephen Mann**, *University of Bristol*
1:40 p.m.	In vitro self-replication and multicistronic expression of large synthetic genomes (PDF)<br>**Hannes Mutschler**, *MPI Biochemistry*
2:05 p.m.	SESSION 3: Emergent Behaviors: From Catalysts to Motility<br>**Chair: Rebecca Schulman**, *Johns Hopkins University*
2:10 p.m.	Enzyme Motors and Pumps: From Transport to Collective Behavior (PDF)<br>**Ayusman Sen**, *Penn State University*
2:35 p.m.	A touch of nonlinearity and heterogeneity in active matter: from swimmers in fluids to mixtures of colloids (PDF)<br>**Daphne Klotsa**, *University of North Carolina Chapel Hill*
3:00 p.m.	Day 1 Ends

+ Tuesday, May 19, 2020

+ Wednesday, May 20, 2020

NANOSCIENCE INITIATIVE
ADVANCED SCIENCE RESEARCH CENTER

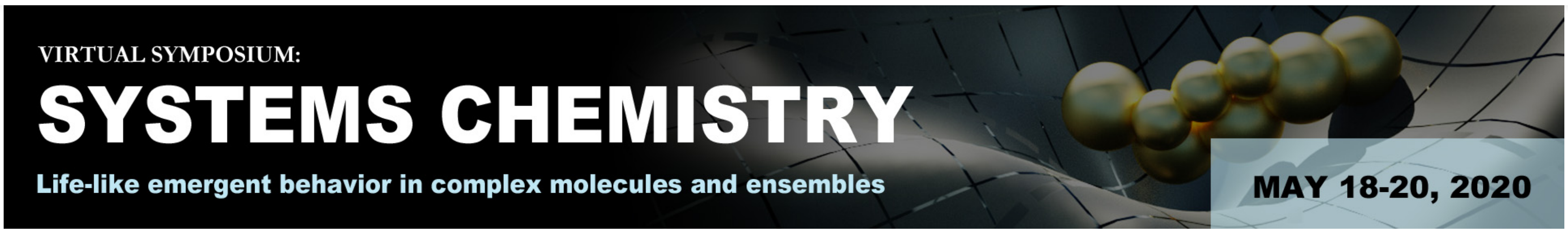

[Home](#)
[Speakers](#)
[Program](#)
[Twitter Poster Session](#)
[Information for Presenters](#)
[Organizers](#)

Follow the Event @syschem20

Contact Us

nanoscience@gc.cuny.edu

Poster Session Winners

Due to the volume of submissions in the #dynamic category, two poster winners were selected.

POSTER PRIZE WINNER #origins Winner

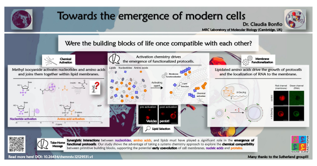

Claudia Bonfio
MRC Laboratory of Molecular Biology, United Kingdom

#dynamic Winner

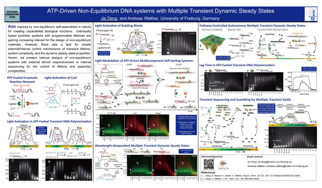

Jie Deng
University of Freiburg, Germany

#bionet Winner

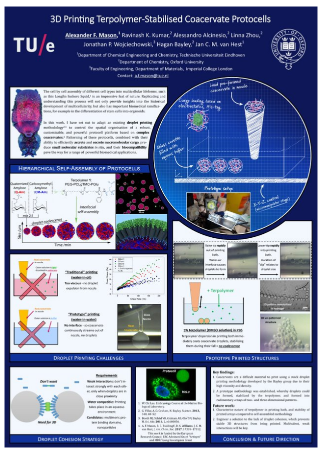

Alex Mason
Eindhoven University of Technology, Netherlands

#emergent Winner

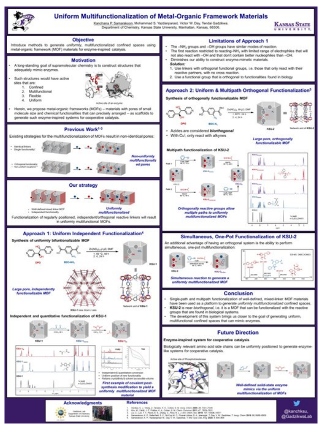

Kanchana Samarakoon
Kansas State University, USA

POSTER PRIZE WINNER #active Winner

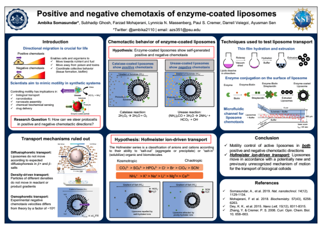

Ambika Somasundar
Pennsylvania State University, USA

#dynamic Winner

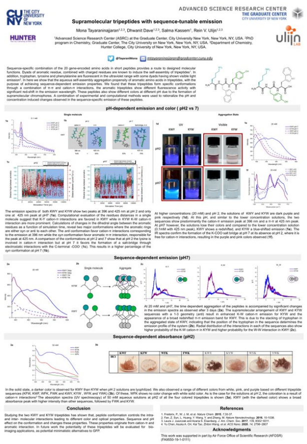

Mona Tayarani Najjaran
Advanced Science Research Center, USA

Honorable Mention: Most "Liked"

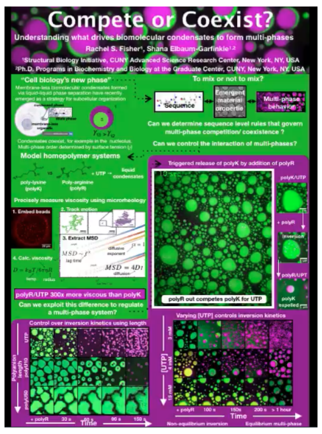

Rachel Fisher
Advanced Science Research Center, USA

Honorable Mention: Most "Liked"

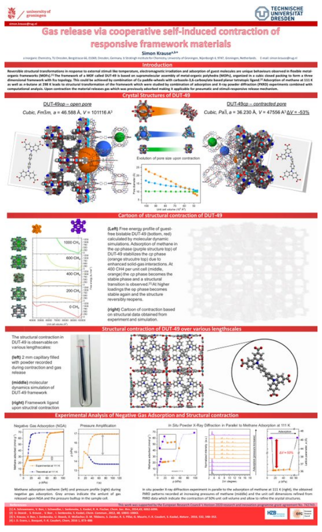

Simon Krause
University of Groningen, Netherlands

Poster Presenters

– Dynamic Information of Molecular Assemblies

Accelerated catalysis in self-assembled states for accessing functional non-equilibrium gels<br>[Subhajit Bai](#), *IISER Kolkata*
Tunable DNA origami motors<br>[Alisina Bazrafshan](#), *Emory University*

(Self)-Sort the Fibers from the Foldamers: two unique supramolecular structures emerge and co-exist from a complex mixture<br>Meagan Beatty, *University of Groningen*
Aryl Boronate Ester Mediated Dynamic Stimuli Responsive G-quadruplex Hydrogels as Drug Delivery Systems<br>Ankan Biswas, *Indian Institute of Technology Indore*
Control of intrinsically disordered phase transitions<br>Robert Devin Bog, *Emory University*
Supramolecular assembly of carbohydrate amphiphiles for biomedical applications<br>Alexandra Brito, *University of Minho*
The Effect of Hydration on the Structure and Dynamics of Phase Separated Biomimetic Cell Membranes<br>Madhurima Chattopadhyay, *Poznan University of Technology*
Molecular Dynamics Force Field Investigations for Monounsaturated Triacylglycerides<br>Robert Cordina, *University of Strathclyde*
Regulating chemically fueled peptide assemblies by molecular design<br>Kun Dai, *Technical University of Munich*
Temporal control of DNA-based nanodevices and DNA-based nanostructure assembly<br>Erica Del Grosso, *University of Rome*
ATP-Driven Non-Equilibrium DNA systems with Multiple Transient Dynamic Steady States<br>Jie Deng, *University of Freiburg*
Dissipative catalysis with a molecular machine<br>Stephen Fielden, *University of Manchester*
Dynamic Covalent Metathesis in the C=C/C=N Exchange between Knoevenagel Compounds and Imines<br>Ruirui Gu, *Supramolecular Science and Engineering Institute*
Tweaking the self-assembly of amyloid-like peptide fibers to hierarchical functional materials<br>Deepika Gupta, *Institute of Nano Science and Technology (INST), Mohali*
Time-Dependent Switching of Constitutional Dynamic Libraries and Networks from Kinetic to Thermodynamic Distributions<br>Meixia He, *Supramolecular Science and Engineering Institute*
Pathway Selection in Charge-complementary Peptide Co-assembly<br>Greg Hudalla, *University of Florida*
one- and two-component organogels containing cyanostilbene without any auxiliary substituents<br>Jagadish Kumar Katla, *Indian Institute of Technology Gandhinagar*
2D Self-Assembly and Reactions in Surfaces<br>Henning Klaasen, *Westfälische Wilhelms – University of Münster*
Unravelling topology-induced shape transformations in dendrimersomes<br>Nina Kostina, *DWI Leibniz Institute for Interactive Materials*
Self-cleaving N-aryl amides: Solvent and temperature dependent equilibrium<br>Vitaly Kovalenko, *Belarusian State Pedagogical University*
Gas release via cooperative self-induced contraction of responsive framework materials<br>Simon Krause, *University of Groningen*
Enzyme-mediated dynamic system allows light-controlled selective formation of cyclodextrins<br>Dennis Larsen, *Technical University of Denmark*
Iodination-promoted strategy for the in-situ generation of selfassembled chiroptically-active gold-peptide superstructures<br>Alessandro Marchetti, *Polytechnic University of Milan*

Automated stirring device for continuous stirring while sampling inside ultra pressure liquid chromatography system<br>Omer Markovitch, *University of Groningen*
Cross-linkable Peptide-based Hydrogels<br>Libby Marshall, *University of Glasgow*
Rotaxane dynamics: quantification of macrocycle shuttling rate<br>Vicente Marti-Centelles, *University of Bordeaux*
Templated Synthesis of alpha-glucans<br>Giorgia Masciotta, *Technical University of Denmark*
Three products selective through time dependance oxidative self sorting of imine libraries<br>Thamon Puangsamlee, *University of Houston*
Dissipative self-assembling fibers: from molecular design to properties<br>Jennifer Rodon Fores, *Technical University of Munich*
Development of a new family of fluorinated amphiphiles<br>Marta Rosati, *Polytechnic University of Milan*
Self-Assembly of Nucleic Acid-Peptide Conjugates<br>Hava Sadihov, *Ben Gurion University of the Negev*
Deconvolution of competitive transient species in multivalent chemical fuel-driven assembly<br>Ekta Shandilya, *Indian Institute of Science Education and Research Mohali*
Social distancing in supramolecular assemblies by fueled reaction cycle<br>Nishant Singh, *University of Strasbourg*
Modifying the surfaces of peptide nano fibers utilizing a thiol-thioester exchange<br>Jillian Smith-Carpenter, *Fairfield University*
Directing peptide liquid-liquid phase transitions with dynamic chemical networks<br>Junjun Tan, *Emory University*
Supramolecular peptide with sequence tunable emission<br>Mona TayaraniNajjaran, *Advanced Science Research Center*
Information-based autonomous reconfiguration in systems of interacting DNA nanostructures<br>Grigory Tikhomirov, *California Institute of Technology*
Understanding the assembly mechanism of a phase-separating bacterial ribonuclease<br>Dylan Tomares, *University of Pittsburgh*
Beyond tripeptides – using active learning to search datasets<br>Alexander van Teijlingen, *University of Strathclyde*
Allosteric regulation of anion exchange in an Fe4L6 tetrahedron<br>Larissa von Krbek, *University of Cambridge*
Sugar-driven formation of artificial raft-domains with hierarchical periodic nanoarrays on dendrimersome protocells<br>Anna Maria Wagner, *DWI Leibniz Institute*
Dynamic Soft Materials from Tunable Supramolecular Recognition<br>Matthew Webber, *University of Notre Dame*
Design of Non-Classical Frank Kasper Phases from Sugar-Polyolefin Hybrid Conjugates<br>Charlotte Wentz, *University of Maryland*
Manipulation of coupled equilibria for chain-length control and dilution induced self-assembly in porphyrin supramolecular polymers<br>Elisabeth Weyndt, *Eindhoven University of Technology*
Easy-To-Make Multi-Function Supramolecular Polymers Enabled by A Naturally Tailored Small Molecule<br>Qi Zhang, *University of Groningen*

Dynamic Spatial Formation and Distribution of Intrinsically Disordered Protein Droplets in Macromolecularly Crowded Protocells
Hang Zhao, *University of Bordeaux*

+ Origins and Synthesis of Life

+ Emergent Behaviors: From Catalysts to Motility

+ Active and Adaptive Materials

+ Biological Networks, from Tissues to Oceans

Privacy | Copyright | Accessibility | Contact Us

Copyright © Advanced Science Research Center at the Graduate Center of the City University of New York

85 St. Nicholas Terrace, New York, NY 10031 - Phone: 212-413-3300

In case of emergency, contact: 212-650-7777
Public Safety General Number: 212-650-6911

NANOSCIENCE INITIATIVE
ADVANCED SCIENCE RESEARCH CENTER

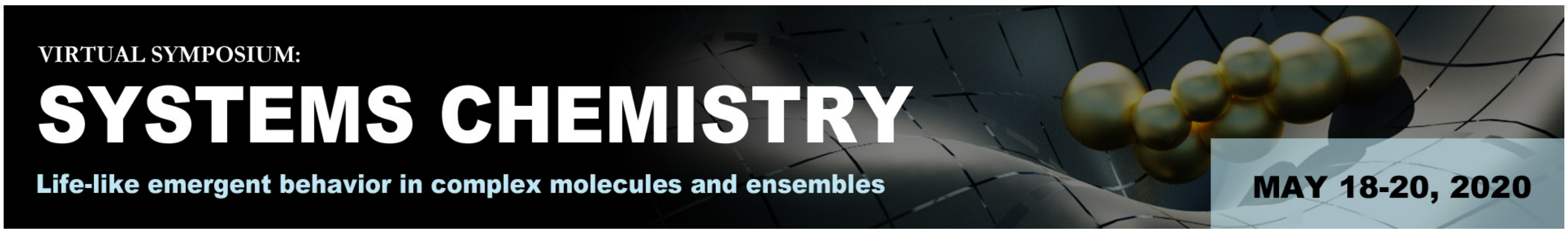

Home
Speakers
Program
Twitter Poster Session
Information for Presenters
Organizers

Follow the Event @syschem20

Contact Us

nanoscience@gc.cuny.edu

Information for Presenters

Present your poster to a global systems chemistry audience via Twitter (@syschem20) for a chance to win an invitation to participate in the 2022 Systems Chemistry Gordon Research Conference with full registration support (worth $1,420 USD).

Poster prize winners will also have the opportunity to present their research talks on the last day of this symposium.

Poster session will start on **Sunday, May 17 at 11:00 a.m. EDT** and commence on **Tuesday, May 19 at 11:00 a.m. EDT**. Winners from the poster session will be selected to present during the symposium on Wednesday, May 20.

Check out our recorded Zoom Info and Q&A Session.

Event Guidelines

1. Set Twitter profile to public for the duration of the poster session event – 11:00 a.m. EDT May 17 to 11:00 a.m. EDT May 19.
2. Tag @syschem20 and use #syschemposter on your official poster tweet. You will be provided with an additional sub-category hashtag to use. Your designated category may not be your first preference.
3. Pin your poster tweet so your poster is easy to find.
4. After posting your official poster, comment on the poster thread and introduce yourself! Feel free to add supporting poster information to the thread as well.
5. Judging will be conducted through the comment thread on your poster by our event judges.
   - Check back here for broad guidelines on judging criteria.
6. Please block and report spurious comment on your poster.

Judging Criteria

1. Research Originality
2. Potential Impact
3. Comprehensiveness
4. Informative
5. Self-Explanatory
6. Design

Poster Guidelines

1. Your poster will be uploaded as an image. JPEG, PNG and GIF are compatible with Twitter.
2. Maximum image size 5 MB.
3. While the recommended image dimensions are 2:1 aspect ratio for landscape, you can use standard poster dimensions (A4, A0) as long as the content is legible (font size 12-16 for A4 and 50-60 for A0).
4. Please be mindful of appropriate permissions while presenting copyright images.
5. Please be mindful of prospective journal policies before presenting unpublished data. Responsibility for the data rests on the presenter.

Rules of Conduct

1. Organizers will not tolerate inappropriate behavior and online harassment of any kind.
2. Comments that demean another person due to his or her gender, gender identity or expression, race, religion, ethnicity, age or disability will not be tolerated.
3. Review of such allegations will be conducted on a case by case basis. Perpetrators can face temporary/permanent ban from the online symposium. Their Twitter/Zoom accounts will also be blocked/reported.
4. Organizers always expect the participants to abide by this code during the symposium and Twitter poster event. Failure in doing so shall result in a void registration and additional appropriate action.

Virtual Poster Resources

1. Quick guide to creating a Twitter account.
2. Quick guide to attending a poster event on Twitter.
3. Blog by Stuart Cantrill on tips for twitter.
4. Check out Royal Society of Chemistry's 2020 Twitter poster winners (reproduced with permission from @Roysocchem). RSC has conducted a Twitter based poster event every year since 2015 (#RSCposter).
5. Want to create an animated poster? Quick guide to creating GIFs.
6. World is not always a happy place and online events always have a threat of spam – quick guide to navigating spam.

FAQ

- What are the guidelines for poster winners giving talks on the final day?
  - If chosen as a poster winner to present on the final day, you will be provided with information on May 19 at 12:00 pm EDT.

- When will the posters be judged?
  - The judges will look throughout the poster session depending on their timezones starting May 17 at 11:00 am EDT until May 19 at 11:00 am EDT. Please ensure that your poster is available for the entirety of the poster session. Please do not delete and repost your posters during this time period.

- What if we interact with participants/judges outside of Twitter? Would that count towards the judging?
  - Interactions with judges outside Twitter will not count towards the judging.

- Will we be giving poster "presentations", or will our poster only be judged visually?
  - Interactions and conversations will take place via comment, likes and retweets on Twitter. There will be no traditional oral component to the poster presentation.

- How will I answer judges' questions, as we may be in different time zones?
  - Feel free to answer questions in your own time; you do not need to respond immediately. Do ensure, however, that you try to address all questions before the conclusion of the poster session.

- What is the registration deadline?
  - The registration deadline is Monday, May 4th, 12:00 pm EDT.

- What if I want to present a poster, but I forgot to include this information on the registration form?
  - Please re-register on the website.

- How do I obtain copyright permission from a journal?
  - Most journals have links on their webpage to facilitate this process. Go to the paper from which you would like to use an image, and follow the instructions on the webpage to obtain copyright permission. If you are the original author, you can modify your own figures without obtaining copyright permissions. Please acknowledge the original source in your figure caption.

Video Resources »

Question - Ana Pina: Mic off, NOVA University of Lisbon, in your oppinion can cooperative catalysis be related with the observed lag phase at begnining, that is influenced by the self-assembly of the monomers?

Answer – Dibyendu Das: Yes Ana, the cooperative catalysis happens from the aggregated state which is accessed after the self-assembly of the monomers. We observed appearance of burst phase almost simultaneously with the formation of the assembled structures. Further, controls done with shorter chain analogues of the substrate (which are unable to assemble), did not show any burst phase. Similarly, no burst phase was observed when the substrate was added to the post assembled solution of the amphiphile. Hence it is the time required for the self-assembly that is responsible for the observed lag phase.

Question - Charalampos Pappas: mic off, University of Groningen. What about non-equilibrium autocatalysis?

Answer – Dibyendu Das: Great question Babis! Autocatalysis generally gives an essence of positive feedback where formation of one molecule templates the formation of more. Examples of autocatalytic synthetic systems are not rare although examples under non-equilibrium thermodynamics are comparatively less explored. As far as our works are concerned, addition of fuel does lead to activation and assembly but we cannot yet say that activation of one monomer leads to activation of others.

Question - Joe Boekhoven: mic off/ Boekhoven/ TUM/ Could you comment on the feedback in the work of Prins or Eelkema and van Esch. I may miss something, but why you refer to that as negative feedback?

Answer – Dibyendu Das: Thanks for your question Job! By negative feedback we mean the component which helps in the dissipation of the energy which drives self-assembly (activation) of the building blocks in nonequilibrium self assembly (NESA). The feedback in the works presented in the talk originates from the self-assembled structures via cooperative catalysis which subsequently degrades the fuel; hence the feedback is intrinsically generated. However, the works of Prins or van Esch show feedback from exogenous sources already present in the system. For example, in the work of Prins, it is the enzyme potato apyrase which cleaves ATP (thermodynamically activated chemical) that leads to the disassembly of the vesicles. Hence the feedback is from the slow degradation of fuel by the enzyme. In the work of Eelkema & van Esch, the feedback is from the base present in the medium which slowly hydrolyzes the self-assembling higher energy esters.

Question - Elad Arad: mic off/Ben Gurion University, Israel/ when adding the nitrophenol, does it affect the beta-sheet structure of the amyloid derivative?

Answer – Dibyendu Das: Thanks Elad for the question. We did not conduct any separate study to investigate the role of nitrophenol on beta-sheet assembly.

Question - Sandip Chattopadhayay: mic off/ IISER PUNE/ If you use electron donating group instade of using an electron withdrowing group then what will be the effect?

Answer – Dibyendu Das: Thanks Sandip for the question. Instead of nitrophenol, we have used phenol (less electron withdrawing) which resulted in a more stable gel. Hence, we reckon that if we use electron donating group (ca. paracresol), a stable ester and gel with prolonged lifetime will be accessed.

Question - Maheen: How do you define your self-assembled structure to be in kinetic trapped state or dissipative state?

Answer – Dibyendu Das: Great question Maheen. For the non-dissipative kinetically trapped state (non-equilibrium self assembly, NESA), the assembled state is trapped in a local minimum of the corresponding energy landscape and does not dissipate energy. Whereas for dissipative NESA, a constant influx of energy or matter is required to sustain the dissipative non-equilibrium state. When the energy flux is stopped or consumed, the system dissipates energy and returns to equilibrium composition. If energy is added again (refueling), the system again accesses the dissipative non-equilibrium state. In our systems, we could observe that fueling is required to access the self-assembled state which subsequently degrades the fuel. We also observed that refueling results in recycling of the process. Thus we can define our system as dissipative NESA.

NANOSCIENCE INITIATIVE
ADVANCED SCIENCE RESEARCH CENTER

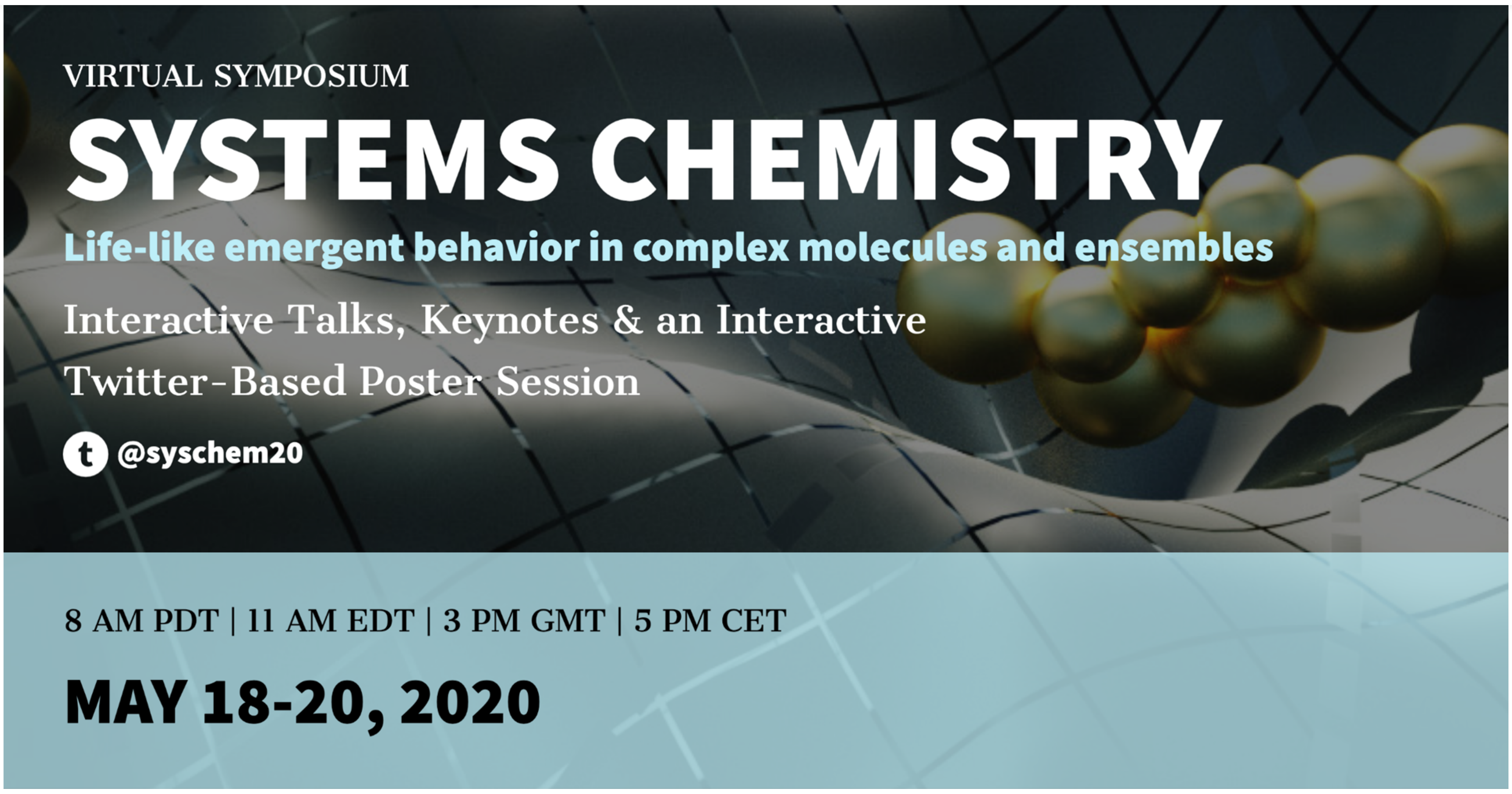

[Home](#)
[Speakers](#)
[Program](#)
[Twitter Poster Session](#)
[Information for Presenters](#)
[Organizers](#)

Follow the Event @syschem20

Contact Us

nanoscience@gc.cuny.edu

Life-like emergent behavior in complex molecules and ensembles

This global virtual symposium will bring together leading scientists in the field of systems chemistry from US, Europe, Asia and Australia to present interactive talks with moderated discussion sessions and a Twitter-based poster session running throughout the event.

This interdisciplinary symposium will cover diverse aspects of the emerging field of systems chemistry, with sessions on:

- Dynamic Information of Molecular Assemblies
- Origins and Synthesis of Life
- Emergent Behaviors: From Catalysts to Motility
- Active and Adaptive Materials
- Biological Networks
- Systems Chemistry and the Coronavirus Crisis

Check out our Participant Guide (PDF) for a summary of our Rules of Conduct, Zoom instructions, and Slack instructions.

Date & Time

This is a three-day virtual symposium, running for four hours each day:

Monday, May 18 – Wednesday, May 20, 2020
8:00 a.m.-12:00 p.m. PDT | 11:00 a.m.-3:00 p.m. EDT | 3:00 p.m.-7:00 p.m. GMT | 5:00 p.m.-9:00 p.m. CET

Slack

Keep the conversation going from the webinar and network with other attendees on our dedicated Slack workspace. Can be accessed through browser or desktop/mobile app. **Join with this invite link.**

You'll automatically be joined to the #welcome channel. Feel free to introduce yourself! Join channels for the different sessions:

- #dynamic: Dynamic Information of Molecular Assemblies
- #origins: Origins and Synthesis of Life
- #emergent: Emergent Behaviors: From Catalysts to Motility
- #active: Active and Adaptive Materials
- #bionet: Biological Networks
- #covid-19: Systems Chemistry and the Coronavirus Crisis
- #posters: Twitter Poster Session
- #random: random things you want to talk about!
- #questions: open questions you have for the organizers

Registration

Registration is closed. Follow us on Twitter (@syschem20) for event updates.

Keynote Speakers

Ben Feringa | *University of Groningen*

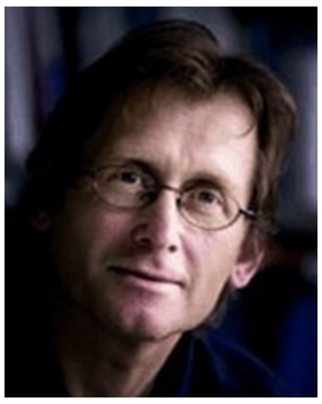

Academic career spanning more than 35 years

Nobel Prize in Chemistry 2016 together with Sir J. Fraser Stoddart and Jean-Pierre Sauvage for the work on Molecular Machines

Notable publications & talks:

- Nobel Prize lecture: The Art of Building Small
- Seminal work on which Nobel Prize work was built: Light-driven monodirectional molecular rotor (*Nature*, 1999)
- Electrically driven directional motion of a four-wheeled molecule on a metal surface (*Nature*, 2011)

Petra Schwille | *Max Planck Institute of Biochemistry*

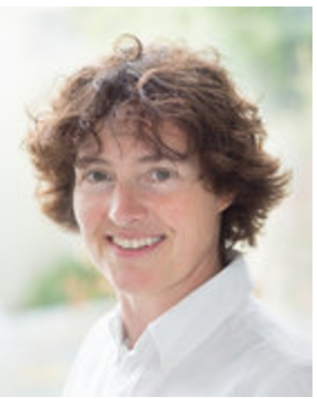

Work has been cited more that 30,000 times

Notable publications:

- Shaping Giant Membrane Vesicles in 3D-Printed Protein Hydrogel Cages (*Small*, 2020)
- Reconstitution of self-organizing protein gradients as spatial cues in cell-free systems (*eLife*, 2014)
- Seminal work on development of fluorescence cross-correlation spectroscopy: Dual-Color Fluorescence Cross-Correlation Spectroscopy for Multicomponent Diffusional Analysis in Solution (PDF) (*Nature*, 1999)

Jenn Heemstra | *Emory University*

Work ranges from biosensing and bioimages to self-assembly

Committed to fostering diversity and inclusivity and blogs regularly on mental and social issues in academia

Notable publications:

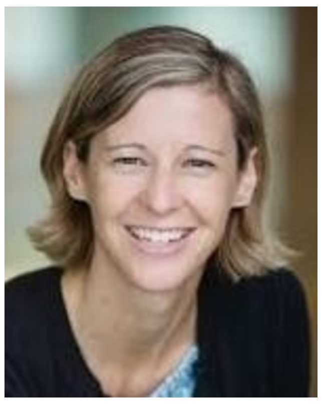

- Peptide nucleic acids harness dual information codes in a single molecule (*Chemical Communications*, 2020)
- Bilingual Peptide Nucleic Acids: Encoding the Languages of Nucleic Acids and Proteins in a Single Self-Assembling Biopolymer (*Journal of the American Chemical Society*, 2019)
- Recent column in Chemical and Engineering News on building resilience in times of uncertainty

THE GREATEST URBAN UNIVERSITY IN THE WORLD

Privacy | Copyright | Accessibility | Contact Us

Copyright © Advanced Science Research Center at the Graduate Center of the City University of New York

85 St. Nicholas Terrace, New York, NY 10031 - Phone: 212-413-3300

In case of emergency, contact: 212-650-7777
Public Safety General Number: 212-650-6911
